# Urolithin A: Biosynthesis at a Glance and Biological Properties Supported by Recent Preclinical and Clinical Evidence

**DOI:** 10.3390/molecules31213982

**Published:** 2026-11-04

**Authors:** Anna Chiara Tenuta, Domenico Iacopetta, Alessia Catalano, Francesca Aiello, Jessica Ceramella, Eva Scarcelli, Francesca Luisa Conforti, Daniela Bonofiglio, Maria Stefania Sinicropi

**Affiliations:** 1 Department of Pharmacy, Health and Nutritional Sciences, University of Calabria, 87036 Cosenza, Italy; annachiaratenuta@gmail.com (A.C.T.); domenico.iacopetta@unical.it (D.I.); francesca.aiello@unical.it (F.A.); jessica.ceramella@unical.it (J.C.); eva.scarcelli@unical.it (E.S.); francescaluisa.conforti@unical.it (F.L.C.); daniela.bonofiglio@unical.it (D.B.); 2 Department of Pharmacy-Drug Sciences, University of Bari “Aldo Moro”, 70126 Bari, Italy

**Keywords:** Urolithin A, antioxidant, anti-inflammatory, anticancer, neuroprotective, mitochondria

## Abstract

Urolithin A (Uro A) is a natural bioactive compound biosynthesized by the gut microbiota after the ingestion of foods that are rich in ellagic acid and ellagitannins, such as strawberries, pomegranate, raspberries and walnuts. Uro A is studied in preclinical and clinical studies for its numerous biological activities, including antioxidant, anti-inflammatory, anticancer, cardioprotective and neuroprotective properties, while also possessing a therapeutic potential against metabolic syndrome and mitochondrial-enhancing effects. This narrative review briefly outlines the biosynthesis of Uro A and current evidence on its biological properties and its potential role in mitigating diseases, on the basis of literature searches performed on Google Scholar, Scopus, and PubMed/MEDLINE. In addition, recent clinical trials based on the supplementation with Uro A are summarized herein.

## 1. Introduction

**Figure 1:**
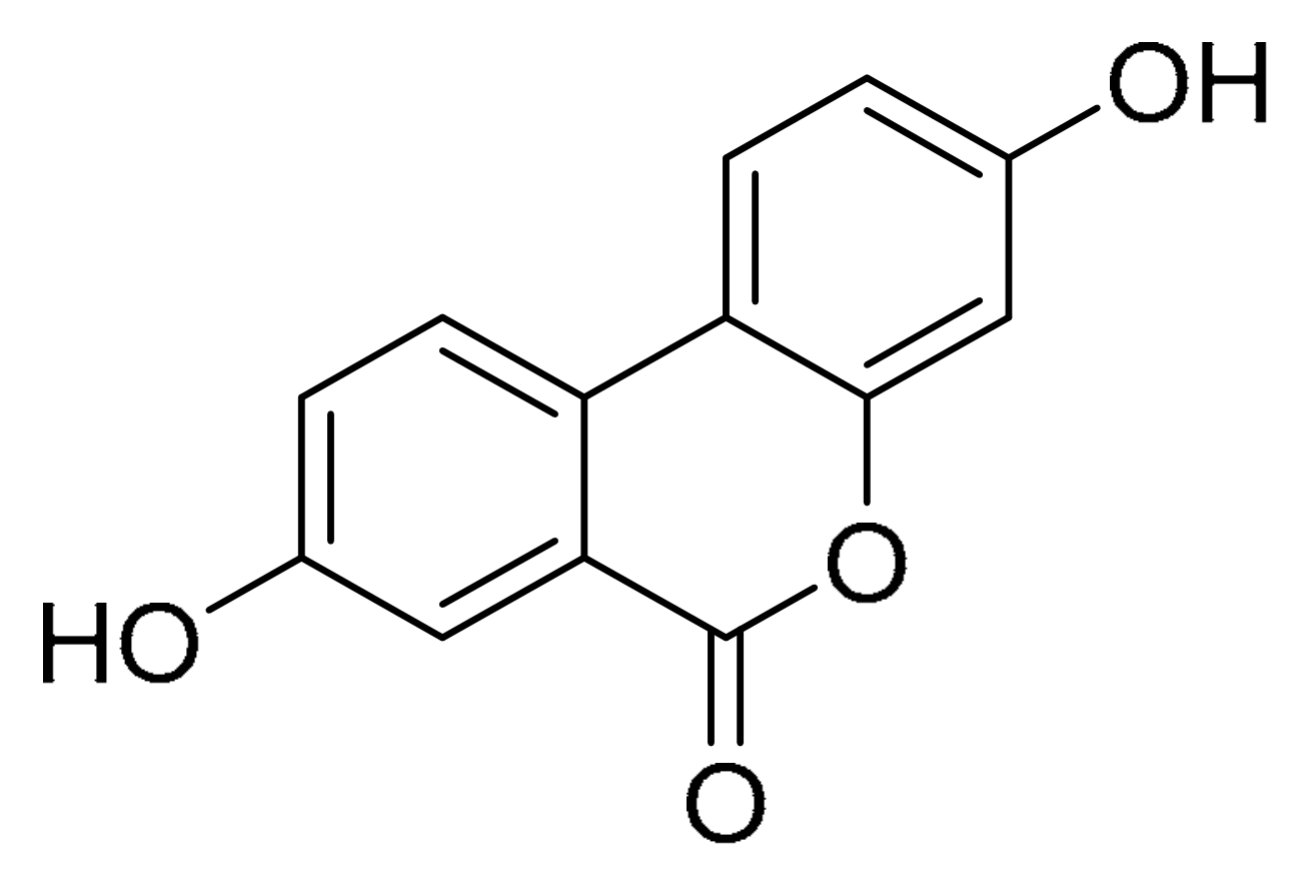
Structure of Uro A.

Urolithin A (Uro A; 3,8-dihydroxy-6*H*-benzo[*c*]chromen-6-one, C_13_H_8_O_4_, Figure 1) is a natural bioactive compound belonging to the category of urolithins, along with Urolithin B (Uro B) and others. Urolithins are obtained through a complex metabolic pathway mediated by the gut microbiota activity after the ingestion of substances like ellagitannins (ETs) and ellagic acid, which are polyphenols occurring in foods like pomegranates (particularly rich in the ET punicalagin), walnuts, almonds, strawberries, raspberries, blackberries, tropical fruits and tea [1,2,3,4]. Contrarily to poorly bioavailable ellagic acid, urolithins are more absorbed and are thought to be responsible for the beneficial health effects attributed to ETs and ellagic acid, found in foods such as berries and dried fruit.

The microbial production of urolithins is characterized by interindividual variability [5]. The composition and functionality of the gut microbiota have been shown to have the largest impact on the metabotypes (i.e., metabolic phenotypes) associated with the biosynthesis of urolithins. However, the metabolic pathway that leads to urolithins is also influenced by numerous parameters, such as sex, age, health, lifestyle, diet and polyphenol consumption. Moreover, ageing and conditions like obesity, metabolic syndrome and pregnancy are often associated with alterations in the gut microbiota, as they all potentially lead to dysbiosis and a decline in the urolithin production [6,7].

**Figure 2:**
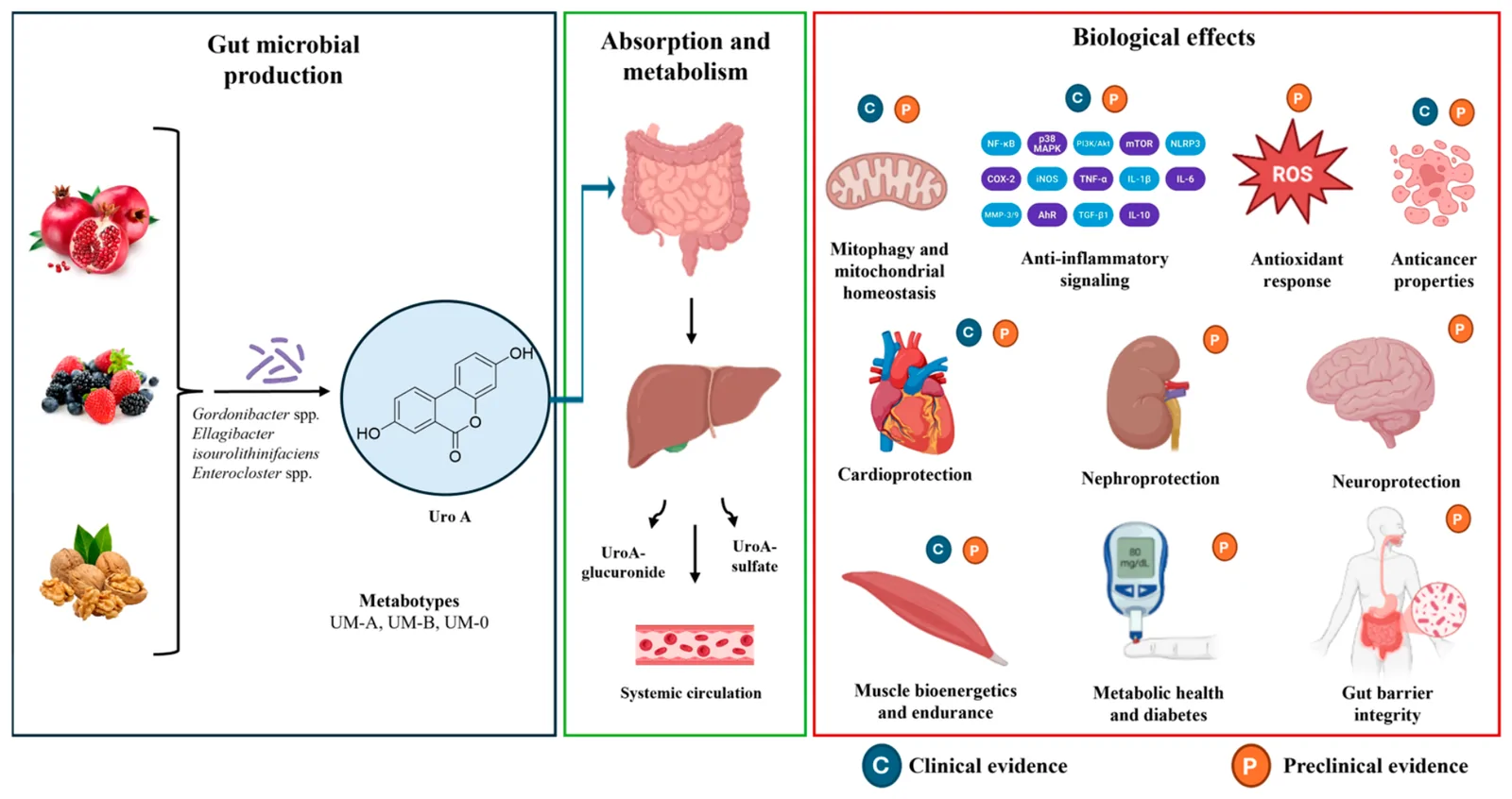
Urolithin A: from dietary precursors to systemic biological effects.

Pharmacokinetic studies revealed that in humans, after the ingestion of foods rich in ellagic acid and ellagitannins, Uro A and Uro B are the predominant and most active metabolites that reach the systemic circulation [8] and are then further processed through phase I and phase II metabolism. However, Uro A is the most extensively investigated metabolite through in vitro, in vivo and clinical studies. Uro A exerts numerous biological activities [9], including antioxidant, anti-inflammatory [10], anticancer [11], cardioprotective [12,13] and neuroprotective properties [14,15,16,17,18]. In addition, it plays a key role in mitigating age-related diseases [19,20] and anxiety due to its mitochondrial-enhancing effects [21,22,23], which are also involved in the enhancement of muscle endurance [24,25], and also possesses therapeutic potential against metabolic syndrome [26]. A recent study demonstrated a potential anti-atherogenic activity of Uro A through the inhibition of pro-atherogenic mechanisms in macrophages and endothelial cells and the alleviation of atherosclerotic plaque burden and inflammation [27]. The potential role of Uro A in mitigating atherosclerosis was also supported by its antiplatelet activity, since enhanced platelet aggregation is one of the hallmarks of this disease [28]. For this reason, supplementation of Uro A could be beneficial in many conditions, although it is affected by poor bioavailability like most natural substances, which represents one of the main clinical limitations. In particular, only a small fraction of Uro A is detected in plasma in the free form, whereas the remaining fraction is further metabolized, mainly through glucuronidation and sulfation. However, Uro A glucuronide and Uro A sulfate have been reported to exert lower in vitro activity than free Uro A, and their biological activities have not been fully elucidated yet [1,3]. In this narrative review, we report the biosynthesis of Uro A and the biological activities exerted by this compound (Figure 2), focusing on the most recent studies. Both preclinical and clinical studies will be described herein.

## 2. Methods

This review aims to provide a detailed overview of Uro A, with particular emphasis on its biosynthesis, the relevance of its supplementation, and its numerous biological properties, supported by preclinical and clinical evidence. Literature research was conducted using the Google Scholar, Scopus and PubMed/MEDLINE search engines, using keywords such as “Urolithin A”, “biological properties of Urolithin A”, “biological activities of Urolithin A”, and “biosynthesis of Urolithin A”. The search was conducted periodically from December 2025 to September 2026, and it was focused especially on studies published within the previous five years. This time frame was selected to ensure the inclusion of the most recent available evidence, given that Uro A is currently one of the most extensively investigated compounds in both preclinical and clinical research. All abstracts and full-text articles were examined for their pertinence to this review. 

Studies were included if they met the following criteria:

a. Original peer-reviewed research articles (in vitro, in vivo animal models, and human clinical trials) published in English with accessible full text;
b. Studies investigating Uro A biosynthesis, pharmacokinetic parameters, metabolic phenotypes (UM-A, UM-B, and UM-0), or molecular mechanisms of action;
c. Studies evaluating Uro A biological activities across diverse organ systems (e.g., cardiovascular, central nervous system, renal, gastrointestinal, and skeletal muscle).

Studies were excluded if they:

a. Focused exclusively on other urolithins (e.g., Uro B or Uro C) or synthetic derivatives without direct relevance or comparative data for native Uro A;
b. Consisted of non-peer-reviewed literature, editorials, conference abstracts lacking full text, or master’s/doctoral dissertations;
c. Presented duplicate data or substantially overlapping findings without providing novel mechanistic insights.

Furthermore, to obtain a broader overview, for each biological property studies employing different experimental models and involving different organs or systems were preferentially included. For in vivo research, studies using validated animal models with standardized Urolithin A dosing and proper control groups were also included. To avoid redundancy, studies reporting substantially overlapping findings were excluded, giving preference to the most comprehensive or recent studies. Moreover, studies offering comprehensive signalling pathways in cellular models and direct molecular target verification in animal models were prioritized over purely observational or single-endpoint reports. To emphasize the importance of Uro A supplementation and to identify correspondences between preclinical findings and evidence in humans, clinical studies supporting the examined biological properties, when available, were also included. In addition to this, a more comprehensive and accurate search was carried out by consulting the ClinicalTrials.gov database (https://clinicaltrials.gov), which was accessed on 19 September 2026. In particular, clinical studies were included if they evaluated oral Uro A supplementation, precursor-rich dietary sources or combination formulations across healthy participants as well as specific clinical and lifestyle populations. Eligible study designs comprised randomized controlled trials (double-blind, triple-masked, or open-label), crossover bioavailability studies, parallel-arm pharmacokinetic profile assessments, and phase 2/3 interventional trials evaluating endpoints related to skeletal muscle energetics, mitochondrial quality, vascular endothelial function, glucose metabolism, or disease-specific biomarkers.

Finally, to ensure the inclusion of robust scientific evidence, a simplified quality assessment framework was applied during the screening process. The selected studies were evaluated based on five core criteria: (1) appropriate control groups, (2) dose- and time-dependent evaluations at physiologically relevant levels, (3) mechanistic validation, (4) methodological reporting, and (5) statistical transparency. Only studies fulfilling the criteria for high evidence quality (4–5 criteria) or moderate evidence quality (3 criteria) were selected for inclusion in this review.

Overall, 109 articles met the inclusion criteria and were cited in this review.

## 3. Urolithins

**Figure 3:**
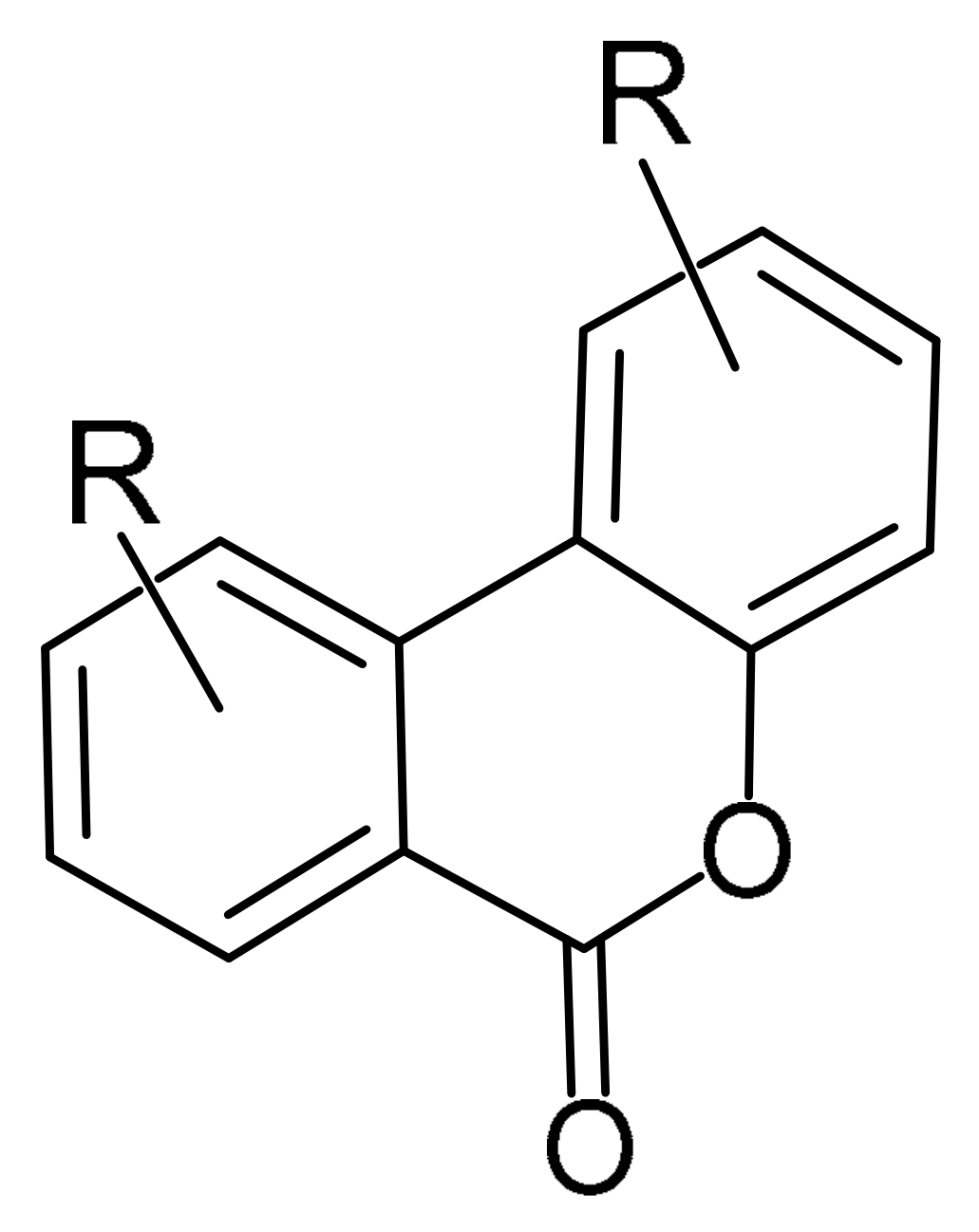
General structure of urolithins.

Urolithins are dibenzo[*b*,*d*]pyran-6-one derivatives and are thus characterized by benzo-coumarin scaffolds with different hydroxylation patterns (Figure 3, R = H, OH, OCH_3_, etc.).

**Figure 4:**
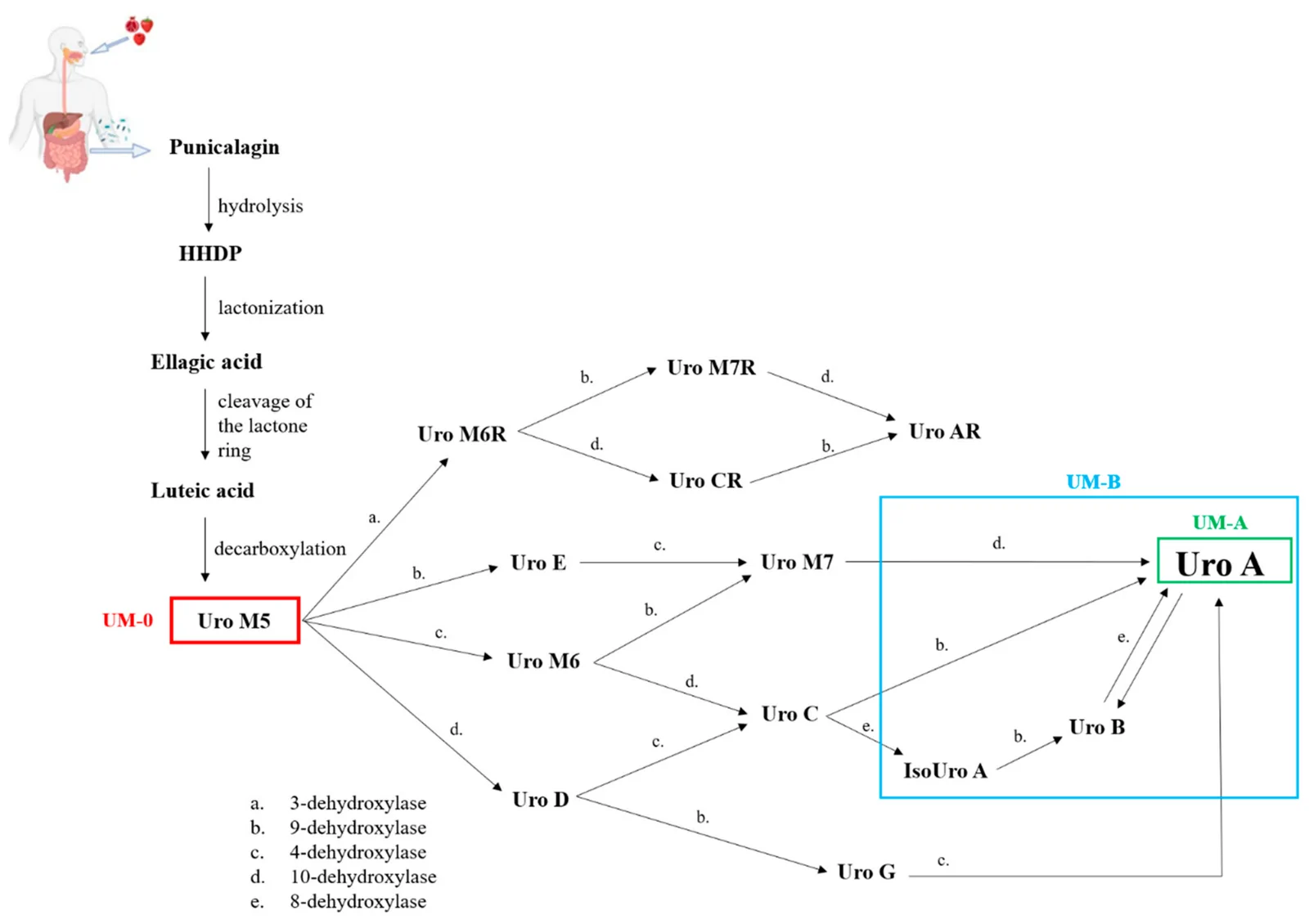
Metabolic pathway for the conversion of ETs (i.e., punicalagin) after the ingestion of ellagitannin-rich foods.

The microbial conversion of ETs to different urolithins, and finally to Uro A, is depicted in Figure 4. Punicalagin is taken as an example of ETs; the structures of the compounds depicted in the figure are summarized in Table 1. Briefly, ETs are hydrolysed to the intermediate hexahydroxydiphenic acid (HHDP) [29] in the stomach and in the small intestine [1] by tannases [3]. Afterwards, through spontaneous lactonization, HHDP converts into ellagic acid, which is poorly bioavailable. The unabsorbed moiety of ellagic acid in the gastrointestinal (GI) tract is then processed by the gut microbiota in the colon [30], mainly in its distal region [31]; through the cleavage of the lactone ring, ellagic acid is converted into luteic acid, which is then decarboxylated to form Uro M5, a pentahydroxy-urolithin. Uro M5 is then dehydroxylated at different positions to form Uro M6R, Uro E, Uro M6 and Uro D (tetrahydroxy-urolithins). Tetrahydroxy-urolithins are converted into Uro M7R, Uro CR, Uro M7, Uro C and Uro G (trihydroxy-urolithins); the latter yield dihydroxy-urolithins (Uro A, IsoUro A and Uro AR) through one- or two-step dehydroxylation; eventually, dihydroxy-urolithins can be further dehydroxylated to monohydroxy-urolithins (IsoUroA → Uro B) [6].

## 4. Gut Bacteria Involved in the Production of Urolithins

A few gut bacteria involved in the production of urolithins have been identified (Table 2). Particularly, it was reported that lactic acid bacteria belonging to the *Lactobacillus* spp. (*L. plantarum*, *L. paraplantarum*, *L. pentosus*, *L. rhamnosus* and *L. casei*) use tannases to convert ETs into ellagic acid [2,32]. The latter is then metabolized into luteic acid, Uro C, Uro M5 and Uro M6 through the enzyme complex of species like *Gordonibacter urolithinfaciens* and *Gordonibacter pamelaeae*. These species belong to the *Eggerthellaceae* family [33,34], the same as the genus *Ellagibacter*, whose bacteria can convert ellagic acid into IsoUro A, Uro C, Uro M5 and Uro M6 [35]. Moreover, the *Gordonibacter* and *Ellagibacter* genera can dehydroxylate Uro D and Uro M5 into Uro M6. Uro A is eventually produced via dehydroxylation of Uro C, which is carried out by the enzyme complex of bacteria of the *Enterocloster* genus, including *E. bolteae, E. asparagiformis*, *E. citroniae*, and *E. pacaense* [33]. Urolithin production in the gut shows a large human interindividual variability. 

Metabotypes (i.e., metabolic phenotypes) are associated with the biosynthesis of urolithins (Figure 3) [6]. Specifically, metabotype A (UM-A) individuals only produce Uro A as a final metabolite, while metabotype B (UM-B) individuals yield Uro B and IsoUro A in addition to Uro A as final products. Finally, metabotype 0 (UM-0) individuals are not able to yield any of these metabolites, though the production of Uro M5, which is not absorbed in the gut, was observed [2,3,36]. The three metabotypes are characterized by different gut microbial abundances. For instance, in UM-A, *Gordonibacter urolithinfaciens* and *Gordonibacter pamelaeae* (family *Eggerthellaceae*) are particularly relevant, together with *Enterocloster* species (*E. bolteae*, *E. asparagiformis*, *E. citroniae*, and *E. pacaense*, family *Lachnospiraceae*) [35,36]. In UM-B, the activity of *E. isourolithinifaciens* and *Enterocloster* species is then predominant, whereas UM-0 individuals display a reduced or insufficient abundance of the above-mentioned genera [35,36], with an overall lower gut microbiome diversity and a reduced *Firmicutes* to *Bacteroidetes* ratio, as demonstrated by faecal microbial analysis in a clinical crossover study in healthy American adults (*n* = 100, aged 18–80 years) [7].

Moreover, the urolithin metabolic pathway is also influenced by sex, age, health, lifestyle, diet and polyphenol consumption. In a large Caucasian cohort (*n* = 839 individuals, aged 5–90 years), urolithin metabotype stratification was performed by analysing first-morning urine via UPLC-ESI-QTOF-MS following a 3-day dietary intake of walnuts, pomegranate juice or pomegranate extract. The percentage of UM-0 individuals was approximately 10%, whereas urolithin biosynthesis declined with ageing, and the persistence of UM-A and UM-B was strictly correlated to age [2,3]. Particularly, about 85% of early-age individuals exhibited UM-A, with this percentage declining to 55% in adults, while 15% of early-age individuals were characterized by UM-B, with prevalence increasing to 45% in adults [2,37]. As ageing and conditions like obesity, metabolic syndrome and pregnancy are often associated with alterations in the gut microbiota, and all of them potentially lead to dysbiosis, they are usually correlated to a decline in the production of Uro A [2,6,7]. Furthermore, a clinical crossover study in healthy American adults (*n* = 100, aged 18–80 years) evaluated urolithin production following the intake of a single dose of 100% pomegranate juice. In this study, Uro A, Uro A glucuronide, and Uro A sulfate were quantified in plasma and Dried Blood Spots (DBSs) using HPLC-MS/MS. It was demonstrated that only 40.8% of the subjects could produce Uro A following the intake of pomegranate juice, confirming that the majority of the adult population lack the microbial capacity for efficient natural conversion [7].

Moreover, in a clinical trial examining adults with varying metabolic profiles (*n* = 119 overall, comprising *n* = 20 healthy normoweight, *n* = 49 healthy overweight/obese, and *n* = 50 metabolic syndrome patients), through targeted UPLC-ESI-qToF-MS metabolomics of 24 h urine and faecal extracts, real-time qPCR quantification of faecal *Gordonibacter* spp., and automated biochemical auto-analyser and Lipoprint subfractionation assays on baseline blood samples, it was observed that individuals that display UM-A are generally metabolic healthy subjects, while individuals with increased cardiometabolic and obesity risk often display UM-B or UM-0. Moreover, a direct correlation of Uro A biosynthesis with high-density lipoprotein cholesterol (HDL-C) levels and positive correlations of IsoUro A and Uro B with total cholesterol, low-density lipoprotein (LDL) cholesterol and oxidized LDL levels were observed [2,38]. However, whether cardiometabolic risk is associated with specific urolithin metabotypes remains to be further investigated.

## 5. Biological Activities of Uro A

Uro A exhibits diverse biological activities, such as anti-inflammatory, antioxidant, anticancer, and anti-ageing properties. It may also promote muscle protein synthesis and muscle growth through several pathways, thus being useful to contrast muscle atrophy. Moreover, it may enhance muscle health and performance by enhancing mitochondrial function and regulating autophagy [39]. These activities are summarized below.

### 5.1. Anti-Inflammatory Activity

Uro A has shown promising anti-inflammatory activity. Recent molecular docking studies revealed that this compound favourably interacts with p38 mitogen activated protein kinase (MAPK) and cyclooxygenase-2 (COX-2) with binding energies that are comparable to anti-inflammatory drugs [1,30]. Moreover, it was demonstrated that Uro A is responsible for the inhibition of MAPK and phosphoinositide 3-kinase (PI3K) activation, impairment of NFKB inhibitor α (IkBα) phosphorylation, and downregulation of pro-inflammatory miRNA, cytokines and mediators in lipopolysaccharide (LPS)-stimulated murine bone-marrow-derived macrophages (Table 3) [40]. Furthermore, studies on rat chondrocytes treated with Uro A reported a downregulation of matrix metalloproteinases, specifically MMP13, MMP3 and MMP9. In addition to this, Uro A suppressed interleukin-1β (IL-1β)-induced expression of inducible nitric oxide synthase (iNOS) and COX-2 and inhibited the IL-1β-induced activation of the MAPK and the NF-κB pathways [41]. According to Bobowska et al. (2021) [42], Uro A inhibited tumour necrosis factor-α (TNF-α) production in LPS-stimulated THP-1 macrophages and, to a greater extent, in human primary macrophages. Moreover, in THP-1 macrophages, Uro A upregulated the expression of TGF-β1, an anti-inflammatory cytokine responsible for activities such as the induction of neutrophil apoptosis and the suppression of the proinflammatory monocyte activation. Uro A’s effects were also extensively studied on RAW 264.7 macrophages, in which the compound was able to decrease NO production and iNOS protein expression in a concentration-dependent manner, while also suppressing pro-inflammatory mediators production through the inhibition of NADPH oxidase (NOX)-derived reactive oxygen species (ROS)-mediated PI3K/Akt/NF-κB, JNK/AP-1 and p38 signalling pathways. Other experiments also confirmed the activity of Uro A on human primary neutrophils isolated from peripheral venous blood, as the compound inhibited the release of azurophilic granules, key components of neutrophil-mediated mechanism of action against pathogenic microorganisms. The effects of Uro A were also investigated in vivo. In an experimental model of osteoarthritis by D’Amico et al. (2022) [43], wild-type adult mice were treated with Uro A for 8 weeks after medial meniscal destabilization surgery. Serum levels of MMP3 were significantly lower, thus leading to an improvement in cartilage degradation in knee joints. Moreover, Uro A was also able to alleviate synovitis, the inflammation of the synovial membrane. Furthermore, Uro A supplementation in *Helicobacter pylori*-infected mice reduced *H. pylori*-induced inflammation, as it reversed IL-10 decline, while downregulating IL-6, IL-8, CXCL1, CCL2 and TNF-α expression [44]. The reduction in IL-6 and TNF-α secretion was also observed in a mouse model of periodontitis, in which Uro A intake promoted collagen fibre reconstruction and repair, while also alleviating bone resorption [45]. The anti-inflammatory activity of Uro A has also been demonstrated in a mouse model of sepsis-induced acute lung injury through the observation of the downregulation of inflammatory markers, such as iNOS, COX-2 and IL-1β [46]. Uro A was also shown to exhibit anti-inflammatory activity in models of airway inflammation, as in an ovalbumin (OVA)-induced acute allergic asthma mouse model the intake of this compound could alleviate pulmonary pathology and airway inflammation. The reported outcomes also involve the inhibition of NOD-like receptor family pyrin domain-containing 3 (NLRP3) inflammasome activity through the EGFR/AKT/FOXO3a phosphorylation pathway [47]. Uro A also has a potential role in mitigating cyclophosphamide-induced premature ovarian failure (POF), which is characterized by microenvironmental oxidative and inflammatory stress. Specifically, in a mouse model of cyclophosphamide-induced POF, the administration of an enhanced formulation composed of Uro A, Coenzyme Q10 (CoQ10), calcium L-5-methyltetrahydrofolate and *Vitex agnus-castus* extract reversed the cyclophosphamide-induced ovarian oxidative–inflammatory shift and thus led to an alleviation of inflammation. This is suggested by the prominent decrease in lipid peroxidation and TNF-α levels and also the decrease in the other pro-inflammatory cytokine levels, such as IL-1β and IL-6. Notably, the administration of the formulation containing Uro A produced more pronounced effects than the one lacking Uro A and than CoQ10 alone [48].

Some clinical studies were also conducted. For instance, a randomized, double-blind, placebo-controlled study performed on an overweight middle-aged population showed that Uro A had an impact on plasma biomarkers of inflammation in a dose-dependent manner. Indeed, the administration of Uro A for a period of 4 months led to a reduction in C-reactive protein (CRP), IFN-γ, IL-1β and TNF-α, even though their levels were already low in these populations [49]. Lower levels of CRP linked to the intake of Uro A were also mentioned by Liu et al. (2022) [50] in a randomized clinical trial on older adults, and by Zhao et al. (2024) in a randomized, double-blind, placebo-controlled study conducted on male athletes [51]. A reduction in IL-2 plasma levels was then reported by Denk et al. (2025) [52] in a randomized, double-blind, placebo-controlled proof-of-concept trial regarding Uro A supplementation for 4 weeks in healthy middle-aged adults. The latter study also described a downregulation of the activation markers CD69 and CXCR4 and, as a consequence, the presence of a less inflammatory NK cell phenotype, together with a reduction in human leukocyte antigen-DR isotype expression, which indicates a less inflammatory classical monocytes phenotype [52].

### 5.2. Antioxidant Activity

According to Marchese et al. (2023) [53] who analysed kinetic and thermochemical studies in aqueous environments, Uro A exerts a greater scavenging activity of the hydroperoxyl radical (•OOH) with respect to known antioxidants like Trolox and melatonin. Uro A’s radical scavenging activity was further validated through chemical tests, such as Oxygen Radical Absorbance Capacity (ORAC), 2,2-diphenyl-picrylhydrazyl (DPPH)-scavenging and superoxide-scavenging tests (Table 4) [54]. Uro A’s antioxidant activity was also evaluated in vitro on Neuro-2a cells subjected to oxidative stress. Specifically, in a dose range of 0.5–4 μM, Uro A was able to mitigate induced oxidative stress, reducing ROS levels and lipid peroxidation, enhancing mitochondrial activity and promoting the activity of superoxide dismutase (SOD), catalase (CAT), glutathione reductase and glutathione peroxidase (GPx) [54]. Furthermore, Uro A’s role as an antioxidant compound was investigated through in vivo studies. For instance, the intraperitoneal administrations of Uro A (50 mg/kg) in hypertensive rats led to a reduction in ROS and serum malondialdehyde and superoxide levels. Moreover, Uro A administration restored the activity of SOD2, usually impaired in hypertension [55]. Uro A also exhibited antioxidant activity in chilled canine spermatozoa. Indeed, Uro A treatment of semen samples enhanced the antioxidant status of spermatozoa, as demonstrated by a reduction in oxidative stress markers (malondialdehyde and H_2_O_2_ levels), an increase in the total antioxidant capacity and the enhanced activity of antioxidant enzymes such as GPx, SOD, and CAT [56].

### 5.3. Uro A and Mitochondria

Mitochondrial health is threatened by senescence and several diseases that cause impaired mitophagy, through which dysfunctional mitochondria are cleared and recycled [57]. It was reported that Uro A is a mitophagy inducer via the activation of PTEN-induced kinase 1 (PINK1)/Parkin-dependent mitophagy [50,57,58] and several other pathways. Upon induction of mitophagy, mitochondrial membrane depolarization leads to the accumulation and stabilization of PINK1 on the outer mitochondrial membrane, with subsequent phosphorylation of ubiquitin and Parkin recruitment and activation. Once activated, the ubiquitin ligase Parkin catalyses the ubiquitination of mitochondrial surface proteins; these ubiquitin moieties are then phosphorylated by PINK1, thus generating phospho-ubiquitin chains. This consequently recruits selective autophagy receptors (such as p62), which via their microtubule-associated protein LC3-interacting domains anchor damaged mitochondria to autophagosomal membranes. Mitochondria are thus engulfed by the phagophore membrane, and the resulting mitophagosome eventually fuses with lysosomes for enzymatic clearance [1,57]. Uro A also activates receptor-mediated (PINK1/Parkin-independent) mitophagy pathways, which occur mainly in tumours or injured tissues [58]. Specifically, mitochondrial membrane receptors such as BNIP3, NIX, and FUNDC1 are characterized by LC3-interacting regions that bind to LC3, thereby promoting autophagosome formation and inducing mitochondrial clearance [57,58]. Other Uro A-mediated pathways correlated with mitophagy include the activation of AMP-activated protein kinase (AMPK), which enhances autophagic flux [1,58], and the modulation of the SIRT3/FOXO3 axis, which promotes mitophagy through the regulation of PINK1/Parkin [1]. Uro A also drives the nuclear translocation of transcription factor EB (TFEB), thereby modulating lysosomal biogenesis and autophagic degradation [1,58].

The mitochondrial enhancing effects were investigated on Uro A-treated canine spermatozoa, where a prevention of the loss of mitochondrial membrane potential and of the decline in oxygen consumption rate and intracellular ATP content were observed [56] (Table 5). Furthermore, evidence suggests that Uro A not only has mitochondrial enhancing effects but also promotes mitochondrial biogenesis through the SIRT1/PGC-1α signalling pathway. Indeed, Uro A treatment on porcine parthenotes led to an upregulation of SIRT1, a key regulator of mitochondrial biogenesis, and PGC-1α, a transcriptional factor. Specifically, once deacetylated by SIRT1, PGC-1α promotes the transcription of nuclear respiratory factor (NRF)1 and NRF2, leading to the synthesis of mitochondrial transcription factor A (TFAM), which is involved in the control of mitochondrial DNA replication [59].

The beneficial effect of Uro A supplementation on mitochondrial health was also investigated through clinical studies. For instance, in a double-blind, placebo-controlled randomized clinical trial involving adults aged from 65 to 90 years, a long-term supplementation of 1000 mg/day Uro A enhanced muscle endurance and reduced biomarkers such as plasma ceramides and acylcarnitine, suggesting an improvement of mitochondrial health [50]. In another trial involving highly trained male distance runners, the administration of 1000 mg/day of Uro A reduced exercise-induced muscle damage and upregulated pathways associated with mitochondrial protein expression [60]. 

### 5.4. Uro A Cardioprotective Activity

Current research suggests that Uro A also exerts cardioprotective properties (Table 6) through mitochondria enhancement [61,62]. For instance, Uro A supplementation was found to reverse doxorubicin-induced interruption of PINK1-Parkin-dependent mitophagy in H9C2 cells and mice, restored PINK1, Parkin, p62 and LC3II proteins levels and upregulated Ambra1 expression, therefore preventing PINK1 degradation and globally ameliorating mitochondrial respiration, with cardioprotective effects [63]. Mitochondrial dysfunction has a major impact on cardiomyocyte bioenergetics, as it is associated with ATP depletion, and, as emphasized by Liu et al. (2025) [64] in a recent study, it can be considered as a hallmark of human heart ageing and disease. In particular, the latter study reports that RNA sequencing confirmed that in a rat model of chronic heart failure with reduced ejection fraction, many pathways associated with mitochondrial respiration and mitochondrial gene sets were affected by heart failure and ameliorated by Uro A. Moreover, enhanced ejection fraction, improved diastolic function, and a reduction in telesystolic volume and ventricle weight following Uro A administration were observed. An improvement in cardiac function was also registered in Uro A-treated old mice through the preservation of the age-related decline in diastolic function (Table 6) [64]. Furthermore, the role of Uro A in myocardial ischemic reperfusion (MIR) was also investigated in a mouse cardiac ischemia/reperfusion (I/R) model. The results showed a decrease in serum lactate dehydrogenase and creatine kinase levels, previously increased by I/R injury, together with a reduction in the myocardial infarct area size and an amelioration of cellular oedema. Furthermore, Uro A was able to attenuate mitochondrial oxidative stress and ferroptosis. These advances suggest that Uro A could protect the myocardium from myocardial ischemia–reperfusion injury [65]. Moreover, Uro A alleviated doxorubicin-induced cardiac dysfunction both in H9C2 cells and C57BL/6 mice, reversing cardiomyocyte contractile dysfunction, myocardial injury and apoptosis. Notably, Uro A increased doxorubicin-induced cardiomyocyte viability and suppressed doxorubicin-induced apoptosis, reversing the elevation of cleaved caspase-3 and BAX and the downregulation of BCL-2 caused by doxorubicin. Furthermore, Uro A partially mitigated damages in cardiac function, induced by doxorubicin-mediated cardiac atrophy, enhanced fibrosis and reduced cell size [63]. The bond between mitochondria and Uro A cardioprotection was also stressed by Huang et al. (2023) [66], who conducted a study on Uro A attenuation of obesity-induced metabolic cardiomyopathy in healthy male C57BL/6 mice. Uro A treatment increased the expression of LC3 and P62 in the mitochondrial fraction of the cardiac tissues, upregulated Parkin and PINK1 genes, increased the occurrence of autophagic vesicles and activated mitophagy in the heart of obese mice. In addition, Uro A administration could ameliorate diastolic dysfunction, decrease cardiac fibrosis and alleviate cardiac hypertrophy [66]. Finally, Uro A was reported to improve vascular endothelial function (VEF) in a placebo-controlled, randomized, double-blinded, parallel group trial. Through the measurement of flow-mediated vasodilatation (FMD) score, indeed, it was found that Uro A administration could improve VEF in non-Uro A producers with poor VEF and lower *Bacillota*/*Bacteroida* ratio [67].

### 5.5. Nephroprotective Properties

According to a few studies, Uro A alleviates renal diseases. For instance, a study on mouse podocytes revealed that Uro A could potentially mitigate diabetic podocytopathy. Specifically, Uro A showed an anti-apoptotic activity through the reduction in ROS levels and an upregulation of autophagic flux in high glucose-treated podocytes. Furthermore, Uro A was found to upregulate nephrin expression and accelerate its turnover, irrespective of ambient glucose concentrations, suggesting that Uro A can also potentially mitigate hyperglycaemia-induced impairment of nephrin expression, which is associated with severe podocyte dysfunction (Table 7) [68]. Another mechanism by which Uro A ameliorates diabetic kidney disease (DKD) involves the induction of mitophagy and anti-inflammatory activity, as demonstrated by Liu et al. (2024) [69] in HK-2 cells and a DKD mouse model. Specifically, in DKD mice, tubulointerstitial fibrosis, F4/80-positive cells and thus macrophage infiltration, increases in mRNA expression levels of pro-inflammatory cytokines and a decrease in mitophagosomes in renal tubular cells and in PINK1 and Parkin expression were observed. Uro A treatment reversed all the mentioned conditions and activated mitophagy, thus alleviating tubulointerstitial inflammation and renal tubular injury. Uro A also may play a role in attenuating calcium oxalate-induced renal injury in kidney stone disease. The adhesion of calcium oxalate crystals to injured tubular epithelial surfaces exacerbates renal damage, as hyperoxaluria triggers ROS production in renal tubular epithelial cells, leading to mitochondrial dysfunction, oxidative stress, and lipid peroxidation. Calcium oxalate monohydrate-stimulated mouse tubular epithelial (TCMK-1) cells and human proximal tubular epithelial (HK-2) cells showed an upregulation of the mRNA expression of tubular injury markers neutrophil gelatinase-associated lipocalin (NGAL), which was reduced by Uro A treatment. Similarly, calcium oxalate monohydrate stimulation upregulated kidney injury molecule-1 (KIM-1) mRNA expression in TCMK-1 cells, which was reversed by Uro A treatment. Furthermore, in a mouse model of renal injury, Uro A reduced the number and area of renal calcium oxalate crystal deposits as well as renal osteopontin levels, a proinflammatory mediator. Uro A also reversed phosphoenolpyruvate carboxykinase 1 (PCK1) downregulation in both mice affected by renal calcium oxalate crystal deposition and calcium oxalate monohydrate-treated cells, thus promoting mitophagy and reducing ROS levels, while upregulating renal PCK1 expression in mice with renal calcium oxalate crystal deposition [70]. Research regarding renal interstitial fibrosis employed a rat model of unilateral ureteral obstruction (UUO) and TGF-β1-treated HK-2 cells. Uro A treatment significantly alleviated renal tissue impairment, decreased macrophage infiltration and downregulated proinflammatory cytokine (TNF-α, IL-6, and MCP-1) expression. Furthermore, Uro A attenuated epithelial –mesenchymal transition by reducing TGF-β1, p-Smad3 and p-38/JNK/ERK, while also increasing the expression of Smad7. As a consequence, Uro A inhibition of the TGF-β1 and MAPK signalling pathways is associated with protective effects on renal fibrosis [71]. However, recently a new mechanism through which Uro A potentially mitigates renal fibrosis was proposed. Specifically, in a UUO-induced fibrosis mouse model, Uro A administration reversed the impairment of mitochondrial fatty acid oxidation and lipid accumulation through GSK3β/β-Catenin signalling negative regulation. Impaired fatty acid oxidation and lipid dysmetabolism were indeed recently identified as hallmarks of renal fibrosis [72]. Furthermore, nephroprotective properties against cisplatin-induced kidney injury were demonstrated through a system of delivery consisting of naringenin-functionalized polyester nanoparticles loaded with Uro A (P2Ns-NAR-UA), which significantly enhanced the bioavailability of Uro A. Specifically, the administration of this formulation in cisplatin-treated mice led to the upregulation of the expression of haem oxygenase-1 (HO-1), therefore alleviating inflammation and oxidative stress and also promoting the activation of the PINK1/Parkin pathway, which is directly involved in the process of mitophagy. Evidence thus suggests that Uro A potentially mitigates cisplatin-induced kidney injury, as the administration of this chemotherapeutic agent is often associated with the induction of oxidative stress, apoptosis and mitochondrial dysfunction in proximal tubular cells [73]. Another study worth mentioning concerns a therapeutic strategy for lupus nephritis based on the synergism between Uro A and cyclosporine A. Lupus nephritis is characterized by inflammation and the accumulation of immune complexes in kidneys, leading to renal damage. Specifically, in MRL-lpr mice, the combined administration of Uro A and cyclosporine A enhanced lymphatic and renal function, as evidenced by lower urinary protein and serum creatinine levels. Moreover, an alleviation of inflammation was observed, as suggested by the reduction in lymphocyte infiltration, IgG deposition in kidneys and in serum chemokine and cytokine levels, such as IL-6, IL-2, IL-10, TNF-α, IFN-γ, CCL19, CCL7, CCL22, CCL3 and CCL1. Notably, while the immunosuppressant agent cyclosporine A alone lacked efficacy and even exacerbated lupus nephritis, and Uro A alone had only a marginal effect, the synergism between the two molecules may represent a potential strategy to mitigate lupus nephritis [74].

### 5.6. Neuroprotective Properties

Uro A could potentially mitigate neurodegenerative diseases. According to a recent computational study, Uro A was found to positively interact with Amyloid-β, acetylcholinesterase and neurotensin receptor, which play a key role in Alzheimer’s disease. Furthermore, as previously mentioned, Uro A is also characterized by a significant binding affinity with COX-2, suggesting this compound’s potential role in mitigating neuroinflammation, which is a hallmark for neurodegenerative diseases, such as Parkinson’s (PD) and Alzheimer’s (AD) diseases (Table 8) [30].

Uro A’s neuroprotective properties were also evaluated both in vitro and on mice exposed to the biotoxin domoic acid (DA), which causes neuronal injury and cognitive deficits through mechanisms like the increase in ROS and mitochondrial dysfunction. Specifically, Uro A treatment was found to exert cytoprotective properties, thus protecting against domoic-acid-induced neuronal death in vitro. Moreover, Uro A administration to DA-treated mice mitigated cognitive impairments through the upregulation of memory-associated proteins, such as NR1 and NR2A, and through mechanisms correlated with Uro A’s antioxidant properties and its protective properties towards mitochondria [75]. Uro A’s ability in modulating neurodegenerative diseases was also demonstrated in other in vivo studies. For instance, Uro A was found to improve learning and memory and, most importantly, to reduce both insoluble and soluble Aβ levels in the prefrontal cortex and total tau levels in mouse models of AD [76]. A recent study highlighted Uro A’s potential activity on the epigenetic aberrations involved in PD using a rotenone rat model of PD. In this study, Uro A administration reversed the rotenone-induced downregulation of SIRT1 at both transcriptional and translational levels, while also increasing DNA methyltransferase-1 (DNMT1) deacetylation without affecting DNMT1 mRNA expression. Additionally, Uro A was able to attenuate alpha-synuclein (SNCA) gene expression, leading to lower α-synuclein protein levels. Collectively, these findings suggest that Uro A potentially modulated the SIRT1/ac-DNMT1/5-mC axis, thereby influencing the global DNA hypermethylation in the substantia nigra and the methylation of the SNCA promoter region [77]. Furthermore, in a sub-acute 1-methyl-4-phenyl-1,2,3,6-tetrahydropyridine (MPTP) PD mouse model, Uro A administration was reported to alleviate cognitive impairment [78]. Moreover, Uro A could reduce MPTP-driven loss of tyrosine hydroxylase (TH)-expressing neurons and striatal dopaminergic nerve terminals, thus alleviating dopaminergic neuronal damage, as confirmed by the presence of higher TH protein levels in Uro A-treated mice than in those only treated with MPTP through Western blot analysis of substantia nigra and striatal extracts. Uro A treatment also alleviated neuroinflammation through the mitigation of MPTP-induced microglia and astrocyte activation and the decrease in pro-inflammatory cytokine levels in the hippocampus of MPTP-treated mice. The same study mentions the activation of the AKT/CREB/BDNF signalling pathway as a pivotal aspect for Uro A’s role in the enhancement of cognitive function, as Uro A administration reversed the MPTP-induced decrease in phospho-AKT protein levels and promoted the phosphorylation of cAMP response element-binding protein (p-CREB) and the expression of brain-derived neurotrophic factor (BDNF) [78]. According to preclinical findings, Uro A modulation of the AKT/CREB/BDNF signalling pathway is also associated with antidepressant-like effects. Indeed, research has shown that the downregulation of p-CREB and BDNF is associated with depressive symptoms. Interestingly, as reported by Di et al. (2025) [79], in a mouse model of chronic social defeat stress (CSDS), Uro A administration decreased the immobility time in both the tail suspension (TST) and the forced swimming tests (FSTs), similarly to the treatment with duloxetine, without any excitatory or inhibitory effects on the central nervous system. Uro A treatment reversed social avoidance and anxious behaviours and increased the expressions of p-AMPK, p-CREB, and BDNF in the mouse hippocampus, thus activating and upregulating, respectively, the CREB/BDNF neurotrophic and the AMPK/CREB/BDNF signalling pathways, with correlated antidepressant-like effects. Moreover, Uro A intake decreased adrenocorticotropic hormone and corticosterone levels, the secretion of pro-inflammatory cytokines, such as IL-6 and IL-1β, in the hippocampus and CSDS-induced increase in p-mTOR and p-AKT, thus alleviating hippocampal inflammation and depression [79]. Uro A also showed a potential beneficial role in a mouse model of alcohol-induced cognitive and social dysfunction (AICSD). Indeed, the administration of Uro A to mice with AICSD led to an improvement in cognitive deficits induced by alcohol consumption, enhancing working memory, short- and long-term memory and sociability. Despite this, evidence suggests that Uro A’s beneficial role is actually preventive rather than therapeutic, as it only exhibited modest effects on long-term alcohol-exposed mice. Moreover, besides mitigating neuroinflammation and synaptic dysfunction as previously reported, Uro A treatment led to an upregulation of the dopamine D_2_ receptor and a downregulation of RAP1, which is pivotal as dopamine signalling has a key role in controlling many brain functions. However, it was demonstrated that Uro A potentially alleviates AICSD through its beneficial action on the gut microbiota and its associated metabolites, and this evidence is supported by the lack of significant effects on memory and sociability in mice treated with antibiotics. Specifically, anandamide (AEA) was proved to mediate Uro A effects on AICSD through the upregulation of DRD2, cannabinoid receptor 1 (CB1R) and the downregulation of RAP1. These preclinical findings suggest that AEA enhances Uro A’s beneficial effects on AICSD through the regulation of the CB1R-DRD2-RAP1 signalling axis [80]. Interestingly, a few preclinical studies also reported that Uro A can potentially mitigate sleep-deprivation-induced cognitive impairment [81,82]. In a murine model of chronic sleep deprivation, Uro A was indeed shown to alleviate anxiety and cognitive deficits through the Nrf2 pathway and the suppression of ferroptosis, while also mitigating neuroinflammation [83]. Uro A-mediated inhibition of ferroptosis and activation of the Nrf2 pathway were also mentioned in a recent study reporting that Uro A mitigated neurological damage in a rat model of middle cerebral artery occlusion/reperfusion [84]. The Uro A-mediated attenuation of neurological damage and cognitive deficits was also evidenced in Cd-exposed mice. Specifically, Uro A administration suppressed Cd-induced activation of the NLRP3 inflammasome in the mouse hippocampus, thus inhibiting neuronal pyroptosis. The Cd-induced activation of the aryl hydrocarbon receptor (AhR) was also suppressed, with subsequent downregulation of the expression of cytochrome CYP1A1, therefore promoting mitochondrial ROS clearance [85]. Another study worth mentioning integrates many of the already-discussed mechanisms in the investigation of Uro A as a potential neuroprotective agent against cyclophosphamide-induced chemobrain in rats. Through its antioxidant, anti-inflammatory, and antiapoptotic activities, together with an influence on acetylcholinesterase activity and the AMPK/SIRT1/PGC-1α/TFAM signalling pathway, Uro A treatment reversed cyclophosphamide-induced cognitive impairment in rats, improving hippocampal histology and decreasing neuronal degeneration [86]. Despite all these findings, Jara et al. (2026) [87] suggest that Uro A is able to reverse cognitive impairment only in middle-aged mice, but not in older ones, supporting Uro A’s preventive rather than therapeutic potential, which particularly relies on mitochondrial enhancement for neuroprotection.

Whether these preclinical findings can be applied to humans is something to be addressed also on the basis of factors like the ability of Uro A to cross the blood–brain barrier (BBB). According to preclinical evidence, Uro A can cross the BBB [1,3] and reach the central nervous system [88]. However, clinical studies are needed to confirm these findings and the ability of Uro A to mitigate neurodegenerative diseases in humans.

**Table 8:** Uro A’s neuroprotective role.

Experimental Model	Dose	Outcomes	Ref.
Molecular docking studies	-	Favourable binding energies of Uro A with Amyloid-β (−5.5 kcal/mol), acetylcholinesterase (−8.8 kcal/mol), neurotensin receptor (−9.9 kcal/mol) and COX2 (−7.97 kcal/mol).	Massaga et al. (2025) [30]
Primary neuron cells previously treated with DA	In vitro: 1, 5 and 10 μM	Increase in survival rates of DA-treated neuronal cells.	Chen et al. (2025) [75]
6-month-old C57BL/6J male mice treated with DA	In vivo: 150 mg/kg for 4 weeks	Upregulation of ERα expression leading to a restoration of NRF-1 expression in the hippocampus of DA-treated mice and thus to an enhancement of NRF-1-driven mitochondrial biogenesis signalling. Reduction in ROS and protein carbonyls levels and suppression of superoxide anion radical generation in the hippocampus. Upregulation of NR1, NR2A, PSD95, and SYN in the hippocampus. Improvement of synaptic function through the alleviation of DA-induced deterioration of neural plasticity. Improvement of cognitive impairments.	
2-month-old male APP/PS1, 12-month-old male and female 3xTgAD and 3xTgAD/Polβ^+/−^ mice	In vivo: 200 mg/kg/day for 5 months	Improvement of learning, memory, and olfactory function in AD mice. Reduction in insoluble and soluble Aβ levels in the prefrontal cortex and total tau levels.	Hou et al. (2024) [76]
9-week-old male Wistar rats (Rotenone rat model of PD)	In vivo: peroral administration of 50 and 100 mg/kg for 40 days (5 days prior to intranigral rotenone infusion and other 35 days postinfusion)	Modulation of the SIRT1/ac-DNMT1/5-mC axis through the following mechanisms: upregulation of the SIRT1 expression and increase in the DNMT1 deacetylation in the substantia nigra of rotenone-treated rats; promotion of 5-methylcytosine (5-mC) levels, and thus global DNA hypermethylation in the substantia nigra and methylation of the SNCA promoter region in the striatum; downregulation of SNCA gene expression and decrease in α-synuclein levels.	Devi et al. (2026) [77]
8-week-old male C57BL/6 J mice (sub-acute MPTP PD mouse model)	In vivo: 20 mg/kg intraperitoneally for 7 days before MPTP injections	Reduction in MPTP-driven loss of TH-expressing neurons and striatal dopaminergic nerve terminals, alleviation of dopaminergic neuronal damage, and mitigation of MPTP-induced microglia and astrocytes activation. Lower levels of pro-inflammatory cytokines, including TNF-α, IL-1β, iNOS, and IL-6, were detected in the hippocampus of MPTP-treated mice. Restoration of phospho-AKT protein levels and promotion of the phosphorylation of p-CREB and the expression of BDNF protein levels.	Xu et al. (2025) [78]
8-week-old male C57BL/6J mice (CSDS model)	In vivo: 50 and 100 mg/kg for 2 weeks	Antidepressant-like activity through a reduction in immobility time in FST and TST, and alleviation of depression-like behaviours, including the avoidance of social interaction and anxiety-like behaviours. Upregulation of the expression of p-AMPK, p-CREB, and BDNF in the mouse hippocampus. Reduction in serum stress hormone levels, hippocampal inflammation, and decrease in the AMPK/CREB/BDNF signalling pathway in the hippocampus of CSDS mice.	Di et al. (2025) [79]
6-week-old male C57BL/6J mice with AICSD	In vivo: 200 mg/kg/day for 8 weeks	Improvement of alcohol-induced cognitive deficits. Enhancement of working memory, short- and long-term memory and sociability. Mitigation of neuroinflammation and synaptic dysfunction. Upregulation of DRD2 and downregulation of RAP1. Uro A effects were enhanced by AEA via the CB1R-DRD2-RAP1 signalling axis.	Zhang et al. (2026) [80]
8-week-old male C57BL/6 mice with induced sleep deprivation	In vivo: 100 mg/kg orally for 2 weeks	Alleviation of cognitive impairments and anxiety and preservation of the hippocampus. Downregulation of the proinflammatory cytokines IL-6, IL-1β, and TNF-α in the hippocampus of mice. Modulation of the Nrf2 pathway with a reduction in Fe^2+^ levels and the suppression of hippocampal ferroptosis.	Wang et al. (2026) [83]
Male Sprague–Dawley rats with middle cerebral artery occlusion/reperfusion (MCAO/R)	In vivo: 30 mg/kg intraperitoneally in two doses: the first one was given at the onset of reperfusion, whereas the second dose was administered 12 h later	Improvement of neurological damages, alleviation of cerebral oedema and blood–brain barrier (BBB) disruption. Decrease in iron accumulation and suppression of ferroptosis. Promotion of Nrf2 nuclear translocation and activation of the Nrf2 pathway.	Gowtham et al. (2026) [84]
6–8-week-old C57BL/6J mice (gender was not specified) with Cd-induced neurotoxicity	In vivo: 200 mg/kg administered daily via oral gavage 2 h prior to intraperitoneal cadmium chloride injection for 8 weeks	Mitigation of hippocampal neuronal/synaptic injury and cognitive deficits. Suppression of Cd-induced activation of NLRP3 inflammasome in hippocampus and inhibition of neuronal pyroptosis. Suppression of Cd-induced AhR and subsequent downregulation of CYP1A1 and promotion of mitochondrial ROS clearance.	Zhu et al. (2026) [85]
Adult male Sprague–Dawley rats affected by cyclophosphamide-induced neurotoxicity	In vivo: 5 and 10 mg/kg intraperitoneally for 10 days	Reverse of cognitive impairment in rats, improvement of hippocampal histology and decrease in neuronal degeneration. Downregulation of the AMPK/SIRT1/PGC-1α/TFAM axis. Increase in the catalase enzymatic activity and glutathione levels. Reverse in the cyclophosphamide-induced increase in NF-κB, TNF-α, P53 and caspase-3 levels, and acetylcholinesterase enzymatic activity.	Labib et al. (2026) [86]

### 5.7. Uro A Gastrointestinal Activity

As described in Section 5.1, Uro A supplementation in *Helicobacter pylori*-infected mice reduced *H. pylori*-induced inflammation and damage [44], but it was found that Uro A also exhibits other beneficial properties in the GI tract (Table 9). For instance, Ma et al. (2023) [89] showed that Uro A treatment alleviated dextran sulfate sodium (DSS)-induced colitis in mice. Indeed, a reverse in weight body loss, an alleviation of diarrhoea and haematochezia, an increase in colon length and an improvement of colon histological parameters were observed. Furthermore, Uro A mitigated gut microbiota dysbiosis and exerted an anti-inflammatory activity, which is pivotal, as inflammation is one of the hallmarks of colitis. Specifically, Uro A treatment was correlated with a decrease in proinflammatory cytokine serum levels and the inhibition of the phosphorylation of the MAPK signalling pathway and activity. Uro A supplementation also reduced intestinal permeability and enhanced intestinal barrier functions with the restoration of the tryptophan catabolites indole-3-aldehyde, indolylpropionic acid, indole-3-lactic acid and L-kynurenine levels. However, Uro A treatment did not significantly reverse DSS-induced decrease in serotonin levels. The enhancement of intestinal barrier integrity mediated by Uro A was also reported in other studies. For instance, research on an in vitro necrotizing enterocolitis (NEC)-in-a-Dish model showed that Uro A improves epithelial barrier function through mechanisms that still need to be investigated and that may not involve junctional protein stabilization. An alleviation of inflammation was also observed through the downregulation of pro-inflammatory cytokine expressions and of NF-κB, NOD-like receptor, TLR signalling and IL-17/Th17, whereas the expression of AhR-dependent genes involved in antioxidant mechanisms was upregulated [90]. Interestingly, the involvement of AhR in the enhancement of gut barrier integrity has also been mentioned in another study. Indeed, Uro A acts a selective ligand for AhR, and in mouse intestinal epithelial cells it was observed that the Uro A-induced AhR activation triggers NLRP6 inflammasome activation, leading to the release of IL-18. This subsequently promotes IL-22 production from innate lymphoid type 3 (ILC3) cells and thus upregulates mucin 2 and regenerating family member 3 gamma. Notably, these findings are consistent with the outcomes of experiments performed on intestinal biopsies of IBD patients, where Uro A treatment increased IL-18 and IL-22 levels. This suggests that Uro A modulates gut barrier function through an NLRP6-IL-18-IL-22 pathway [91]. Moreover, Uro A was shown to prevent inorganic arsenic-induced gut barrier dysfunction. Specifically, in humanized AS3MT mice (in which the mouse arsenic methyltransferase gene, *As3mt*, was replaced with human AS3MT, hAS3MT) exposed to 100 ppb inorganic arsenic for 28 weeks, Uro A ameliorated inorganic-arsenic-induced gut permeability, provided protection from gut dysbiosis and suppressed colon shortening. Furthermore, Uro A treatment decreased inflammatory cytokine levels and myeloperoxidase activity [92]. As reported by Xiao et al. (2026) [93], Uro A’s anti-inflammatory activity can also provide hepatoprotection. Interestingly, Uro A alleviated D-gal-induced liver injury and hepatic inflammation associated with ageing. This can be correlated with the modulation of the hepatic Nur77-MDM2 axis through the stabilization of Nur77 that occurs by inhibiting its ubiquitination. Furthermore, the upregulation of IL-6 and IL-1β was reduced, and IL-10 levels were restored, while D-gal-induced hepatocyte inflammation and structural damage were reversed. Moreover, D-gal-induced dysfunction and dysregulated lipid metabolism were mitigated through a reduction in alanine aminotransferase, aspartate aminotransferase, triglyceride, and total cholesterol levels [93]. 

### 5.8. Uro A’s Effects on Metabolic Syndrome and Diabetes

According to previous research, Uro A represents a potential multi-target compound in metabolic syndrome and diabetes through the modulation of several pathways involved in obesity, dyslipidaemia, and insulin resistance. A recent study conducted by Berköz et al. (2026) [94] on 3T3-L1 cells, which are adipocyte progenitor cells, reported that Uro A treatment was able to inhibit adipogenesis and lipogenesis, while also enhancing lipolysis (Table 10). Specifically, 3T3-L1 cells treated with Uro A showed a concentration-dependent reduction in lipid storage during adipogenesis and a suppression of lipogenesis by reducing glycerol-3-phosphate dehydrogenase activity. Furthermore, Uro A treatment enhanced free glycerol release and thus lipolysis. A downregulation of the transcription factor PPARγ, which is directly involved in adipocyte differentiation, and of C/EBPα and SREBP-1c, responsible for managing fatty acid synthesis and intracellular triglyceride storage, was also reported. The expression of adipocyte fatty-acid-binding protein and fatty acid synthase, which regulate the uptake of long-chain fatty acids into adipocytes and their differentiation, adipogenesis and triglyceride deposition, was inhibited. The mechanism through which Uro A suppresses adipogenesis and lipogenesis, however, also involves the downregulation of the metabolism-regulating adiponectin and leptin genes [94]. Uro A’s beneficial effects on metabolic syndrome and obesity were also investigated by Geng et al. (2025) [95] in high-fat diet (HFD)-induced obese mice, and an amelioration of HFD-induced dyslipidaemia was reported. Indeed, Uro A treatment was found to reduce perinephric and mesenteric fat, with no changes in body weight. Moreover, Uro A administration led to a reduction in triglyceride levels and an increase in HDL-C levels, while also alleviating the systemic inflammation associated with obesity. Uro A was also found to have potential antidiabetic effects by ameliorating glucose intolerance [96] and insulin sensitivity [97]. Indeed, Uro A treatment on L6 myotubes under insulin absence led to an enhancement of glucose uptake through PI3K/Akt and AMPK signalling pathways and to an increase in p-Akt and p-AMPK. It is known that these two phosphorylated proteins play a key role in promoting glucose uptake through GLUT4 translocation from the cytosol to the plasma membrane, which was indeed upregulated by Uro A [96]. This mechanism was also investigated in vivo in a model of type 2 diabetes (T2D) on KK-A^y^/Ta mice. Notably, intraperitoneal glucose tolerance test results after an intraperitoneal glucose injection showed that Uro A prevented the hyperglycaemic effects by blocking the increase in blood glucose levels [96]. The antidiabetic activity was further investigated in type 2 diabetes mellitus (T2DM) mouse models. Specifically, it was reported that not only could Uro A decrease blood glucose levels, but it also had an influence on insulin resistance through the reduction in insulin and TNF-α serum levels and the improvement of the activation of insulin signalling pathway transmission in muscle tissues. As suggested by surface plasmon resonance and molecular docking experiments, this effect may be associated with Uro A-mediated inhibition of TNF-α signalling, as this proinflammatory cytokine can induce insulin resistance [98]. These mechanisms were also investigated on primary human myotubes, where a Uro A-induced upregulation of GLUT4 was observed, which led to an enhancement of basal- and insulin-stimulated glucose uptake [97]. 

### 5.9. Anticancer Properties of Uro A

Scientific studies evidenced the anticancer properties of Uro A both in vitro and in vivo through several mechanisms and in many types of cancer. 

For instance, Remadevi et al. (2024) [99] demonstrated Uro A’s potential role in the treatment of oral squamous cell carcinoma (OSCC) (Table 11). Interestingly, Uro A negatively affected the viability and the proliferation of oral squamous cell carcinoma (SAS and RCB1015) OSCC cell lines, also leading to morphological alterations and shrinkage of cells [99]. 

Further experiments conducted by the same research group clarified that Uro A induces apoptosis via the intrinsic pathway by stimulating ROS production, autophagy and cell cycle arrest in the G2/M or S phase, while also upregulating the p53 and p27 proteins [99].

Moreover, Uro A exerts anticancer properties in OSCC cells through the inhibition of the mTOR/AKT/ERK1/2 pathway and by reducing the metastatic potential [99]. 

In addition to this, in vivo experiments showed that Uro A treatment was able to reduce tumour volume in NOD-SCID mice, without affecting their body weight and with no signs of organ-related toxicity or structural tissue damage, as revealed by histological examination via haematoxylin and eosin (H&E) of major harvested organs (heart, liver, spleen, lungs, kidneys, and brain) conducted at the end of the study [99]. 

Zheng et al. (2025) [100] highlighted the effects of Uro A treatment on tumour-associated macrophages and on breast cancer cell lines MDA-MB-231, BT-549, MCF-7 and 4 T1, showing how a synergistic combination between the anti-inflammatory, mitophagy-promoting and anticancer activity could potentially support breast cancer treatment. Indeed, Uro A showed an antiproliferative activity and an attenuation of migration in breast cancer cells [100]. 

Moreover, experiment results on MDA-MB-231 and BT-549 cell lines also suggest that Uro A inhibited the transcription of genes like c-myc, muc1 and cyclinD1, whereas it downregulated mRNA expression of the proinflammatory cytokines IL-6 and TNF-α and reduced the M2 polarization in a tumour-associated macrophage model. The authors also focused their research on the effect on transcription factor EB (TFEB) in tumour-associated macrophages, which is responsible for the regulation of the biological functions of lysosomes and autophagosomes and at high levels is associated with a better prognosis in breast cancer. Specifically, Uro A treatment was found to promote TFEB’s nuclear translocation, which leads to mitophagy induction, thus preventing cytoplasmic leakage of nucleic acids, which is an event correlated with macrophage inflammation. The combination of all these mechanisms is an important strategy to reduce risk factors for inflammation and tumour progression [100]. 

Another important recent advance concerns Uro A’s potential role in multiple myeloma therapy. Rodríguez-García et al. (2025) [101] investigated Uro A’s activity on the JJN3, U266, RPMI-8226, XG-1 and ARP-1 multiple-myeloma cell lines and reported that the compound showed cytotoxicity in all of these cell lines, while also inhibiting proliferation and inducing cell cycle arrest and increasing p27, BAX, p53 and p21 levels. Ex vivo investigations on primary plasma cells from patients affected by multiple myeloma were also conducted and showed that Uro A treatment reduced CD38+ and CD138+ plasma cells’ viability, without exhibiting cytotoxic effects on peripheral blood mononuclear cells (PBMCs) or non-transformed T cells isolated from healthy donors. Uro A exerts anticancer effects in vivo too, as it was able to inhibit the proliferation of tumour cells in a mouse model of multiple myeloma without reported signs of toxicity during the short-term observation period [101]. 

Common treatments for this cancer, such as bortezomib administration, can only improve long-term survival, as currently there are no adequate strategies able to block tumour progression without the development of treatment resistance. However, the authors also proved that co-treatment of the bortezomib-resistant JJN3-r-Btz cell line and of the equivalent bortezomib-sensitive cells with Uro A and bortezomib exerted a synergistic cytotoxic effect in both cell lines. Furthermore, after the co-treatment, an upregulation of p53 and a downregulation of p-mTOR levels were observed in JJN3-R-Btz cells, together with an upregulation of p21 levels in both the JJN3_r-Btz cell line and the equivalent bortezomib-sensitive cells [101]. 

Emerging evidence also supports the potential of Uro A as a chemopreventive agent in vivo against colorectal cancer (CRC) through the modulation of AKT1-related signalling. Specifically, Uro A enhances immune response through CD8^+^ T-cell infiltration and cytotoxic activation, while also inhibiting the AKT/mTOR signalling pathway and thus tumour growth and proliferation, migration and invasion of tumour cells. Interestingly, Uro A downregulates p-AKT1 phosphorylation without suppressing p-AKT1 in normal colonic epithelial cell lines NCM460 and MCEC [102]. 

A clinical trial was also conducted to investigate the role of walnuts, and specifically Uro A, in cancer prevention, involving 39 patients aged between 50 and 65 years old. The subjects consumed 56 g/day of peeled walnuts and were subject to metabolomic analysis to measure Uro A production levels. These were correlated with an increase in the serum inflammatory marker peptide YY, which inhibits colorectal cancer development, and a decrease in serum sICAM-1 levels, a biomarker for many types of cancer, such as colorectal cancer. Moreover, a downregulation of the expression of vimentin and CD163 in colonic polyp tissue was observed. Overall, these findings suggest the potential role of walnut consumption in subjects with Uro A-producing metabotypes in colorectal cancer prevention [103]. 

## 6. Uro A as a Nutraceutical

### 6.1. Supplementation and Safety of Uro A

Nowadays, natural bioactive compounds have gained popularity for their health benefits and, unfortunately, for the mistaken common belief according to which “natural” means “safe”. However, thanks to their biological properties, nutraceuticals actually represent potential allies in preventing several types of diseases or in combination therapies with pharmaceuticals, where synergistic effects can allow lower therapeutic doses, thus reducing both the incidence and severity of adverse effects. Uro A exerts relevant biological activities, and its supplementation represents a promising strategy to mitigate several diseases associated with hallmarks such as oxidative stress and chronic inflammation and to manage metabolic syndrome and diabetes, as discussed in Section 5. Furthermore, extensive preclinical research and clinical trials describe it as a generally safe and well-tolerated compound, at least at the doses that have been investigated so far [104], although a recent study reported the presence of Uro A-containing stones in the urinary tract of eight individuals (59–87 years) that regularly consumed ET- and EA-rich foods [105]. However, further clinical research is required to confirm the efficacy and safety of Uro A supplementation [106]. Uro A is affected by poor bioavailability, and its biosynthesis is influenced by the composition and functionality of the gut microbiota, which is strictly correlated with factors like sex, age, health, lifestyle, diet, polyphenol consumption, etc. This suggests that the intake of ET- and EA-rich foods alone does not guarantee the production, the absorption and the expression of Uro A’s biological activities. These limitations may be addressed through the development of Uro A delivery systems [73,107,108,109] or with the synthesis of Uro A derivatives, which can be designed and investigated with molecular docking studies not only to improve bioavailability, but also to possibly identify novel derivatives with enhanced or additional biological properties compared with Uro A.

### 6.2. Clinical Trials

Some clinical trials have already been mentioned above in Section 5. Additionally, some other trials have been conducted or are currently in progress. The most recent interventional clinical studies are summarized in Table 12 [110]. 

## 7. Conclusions

Uro A is a gut metabolite derived from ellagic-acid-containing foods such as pomegranates, berries, and walnuts. Its growing interest is due to its therapeutic potential for various diseases. The biosynthesis of Uro A is highly variable and limited by various factors, such as metabolic phenotype, health status and diet of individuals; thus, the intake of ET- and ellagic-acid-rich foods does not guarantee the production, the absorption and the biological activities of Urolithin A. This review outlines the numerous biological properties of Uro A that are still currently under research through preclinical and clinical studies, according to which Uro A may be a suitable candidate for mitigating many pathologies, especially those involving inflammation and oxidative stress, such as age-related diseases and metabolic syndrome. In particular, clinical evidence from randomized controlled trials supports its efficacy in improving mitochondrial health, metabolic parameters, skeletal muscle endurance and physical performance in middle-aged adults. Emerging clinical trials demonstrate promising outcomes regarding vascular endothelial function and immune/gut-barrier regulation. A broad range of organ-protective properties, including neuroprotective, cardioprotective, anti-fibrotic and anti-cancer actions, currently remain grounded in in vitro and in vivo animal models. To date, the conducted preclinical and clinical studies have not identified notable side effects correlated with Uro A supplementation, which suggests that this nutraceutical is well tolerated within the tested dose ranges. This highlights that Uro A supplementation may have a potential preventive role against several diseases, in synergism with pharmaceuticals. This might allow lower therapeutic doses and thereby reduce both the incidence and severity of adverse events. However, the majority of reported studies are preclinical and therefore insufficient to confirm the numerous biological properties associated with Uro A and the absence of relevant side effects in humans. Further clinical research is thus required to establish both the therapeutic efficacy and the long-term safety profile of Uro A, also considering the fact that Uro A has a limited bioavailability, and therefore strategies such as delivering systems must be developed and evaluated.

## Figures and Tables

**Table 1:** Structures of the compounds described in Figure 4.

Compound	Structure	Compound	Structure
Punicalagin	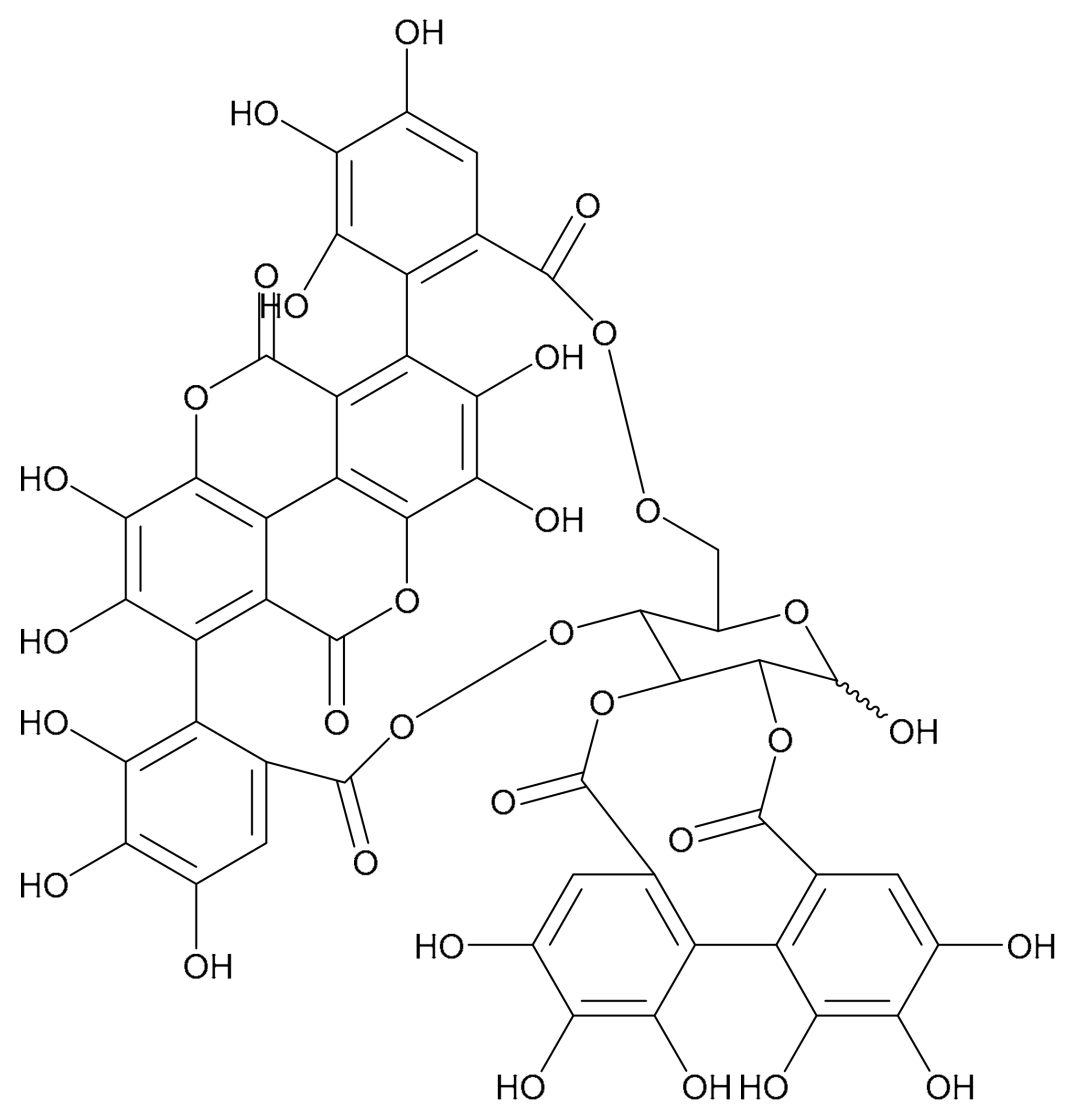	HHDP	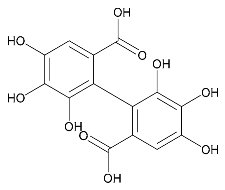
Ellagic acid	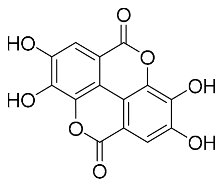	Luteic acid	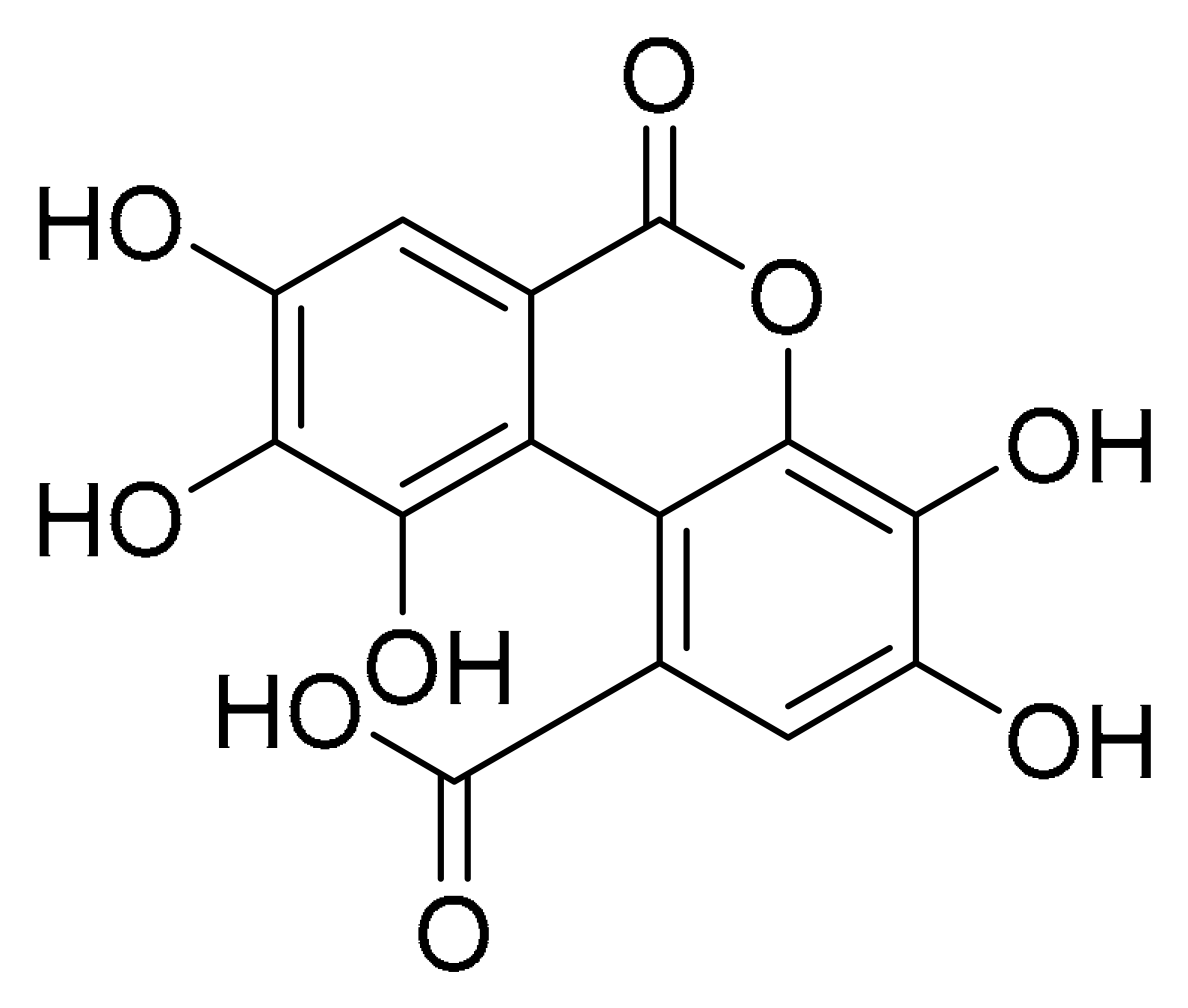
Uro M5	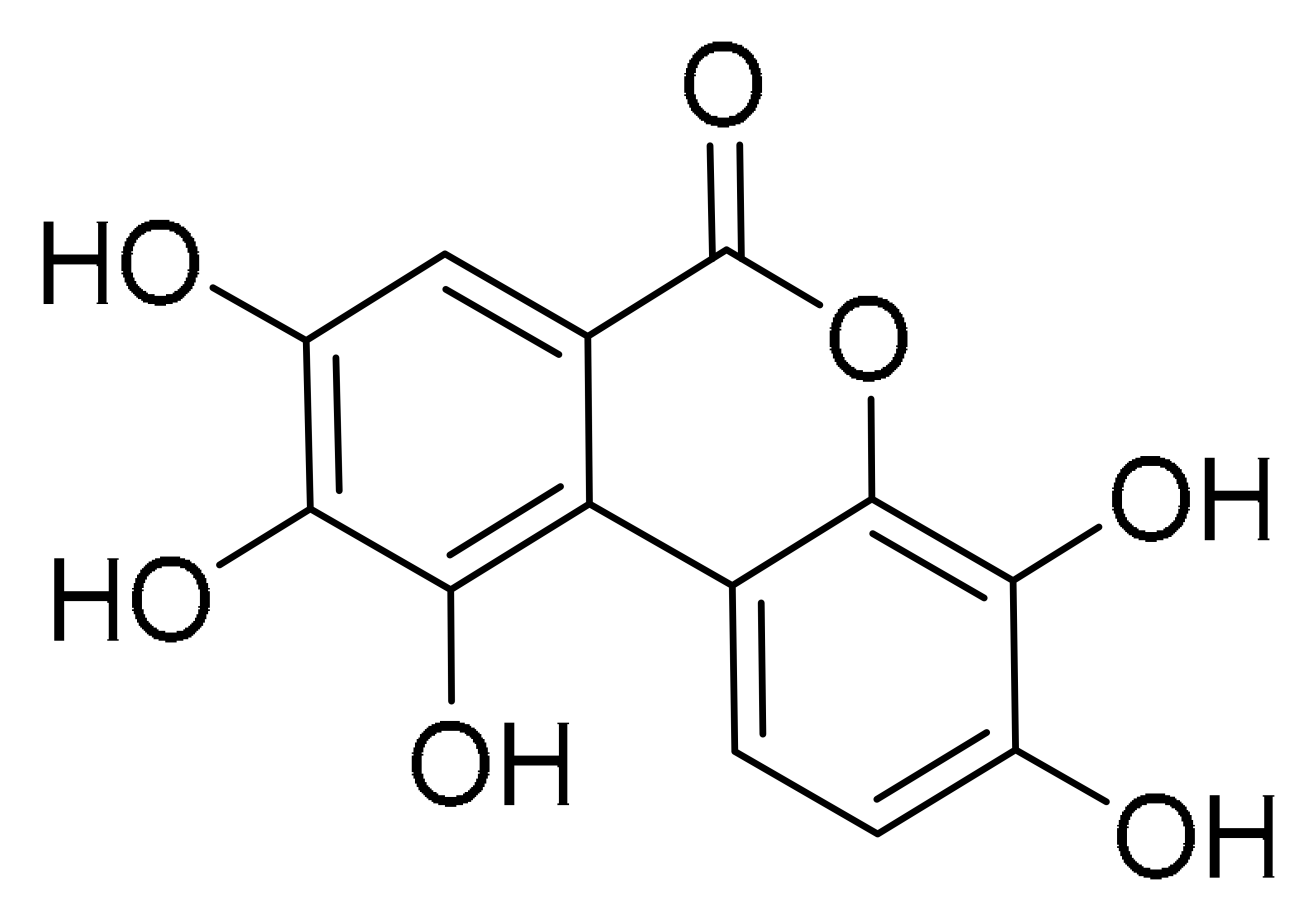	Uro M6R	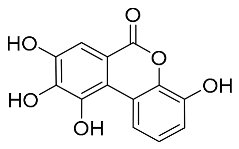
Uro M7R	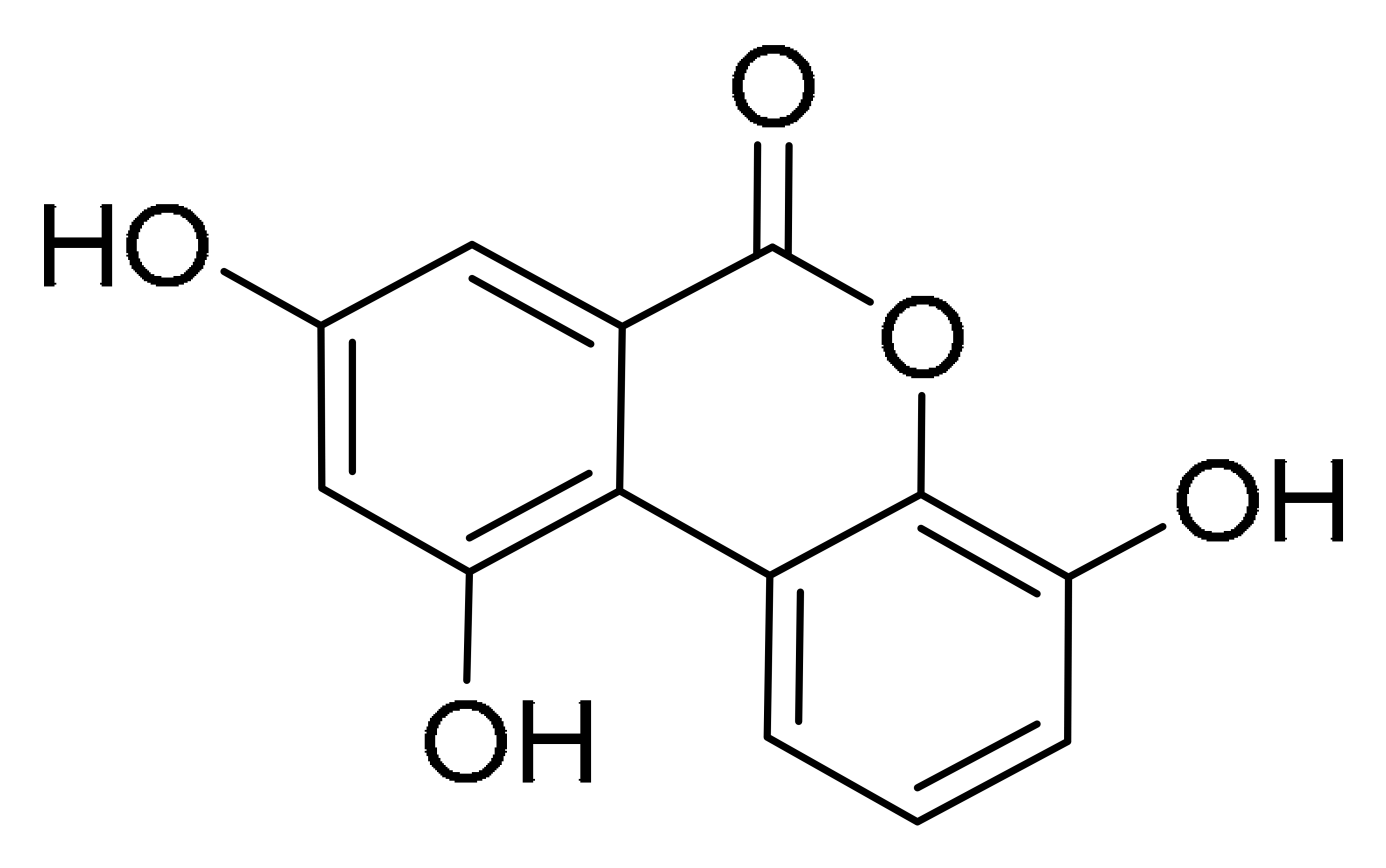	Uro CR	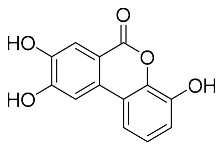
Uro AR	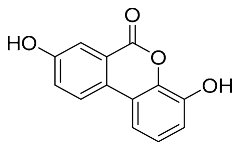	Uro E	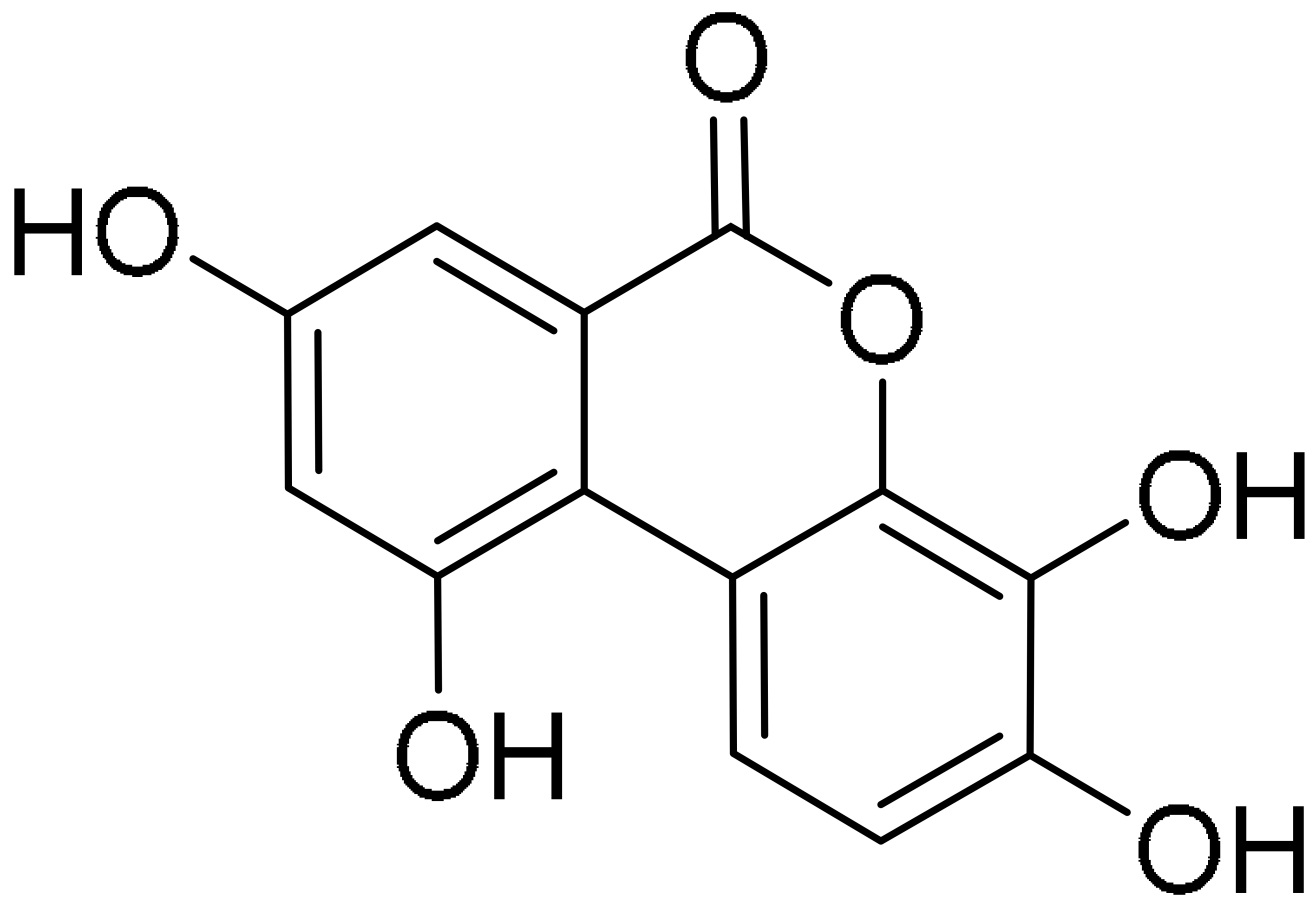
Uro M7	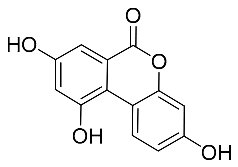	Uro M6	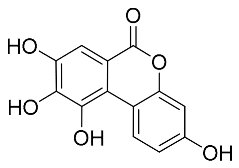
Uro C	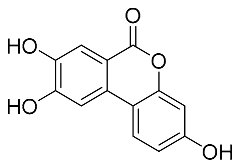	Uro D	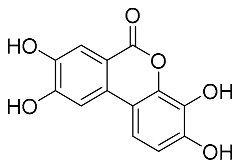
Uro G	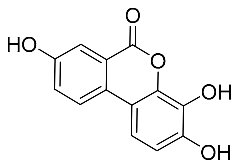	IsoUroA	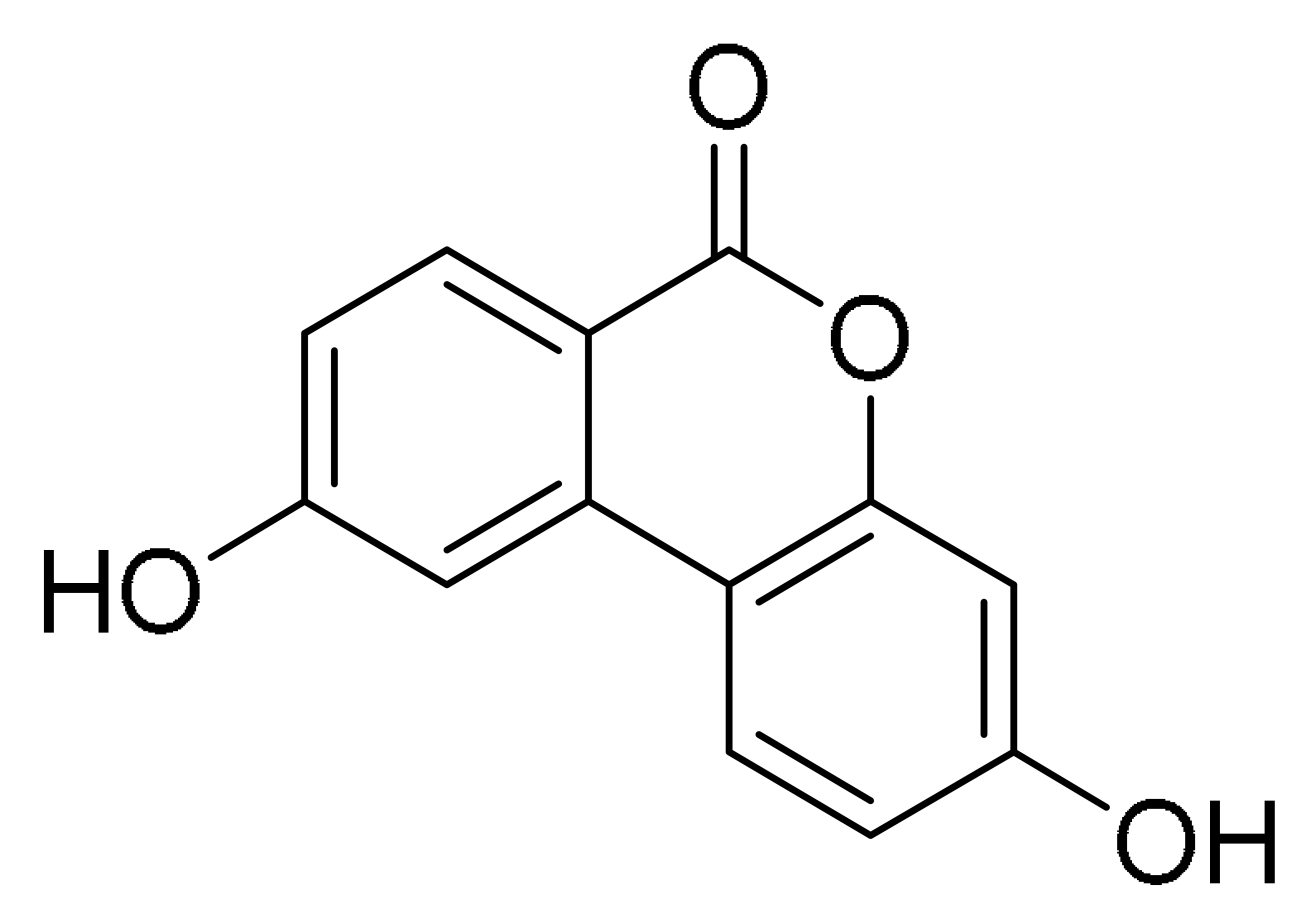
UroB	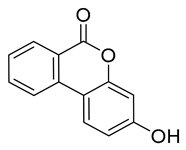	UroA	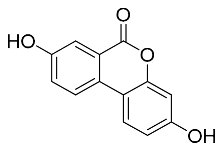

**Table 2:** Summary of the bacterial species and their role in the biosynthesis of urolithins.

Bacterial Species	Key Enzymes	Substrates	Products	Ref.
*Gordonibacter urolithinfaciens* and *Gordonibacter pamelaeae*	Lactonase, decarboxylase, 4-dehydroxylase and 10-dehydroxylase	Ellagic acid Luteic acid Uro M5 Uro M6	Luteic acid Uro M5 Uro M6 Uro C	[35,36]
*Ellagibacter isourolithinifaciens*	Lactonase, decarboxylase, 4-dehydroxylase, 8-dehydroxylase and 10-dehydroxylase	Ellagic acid Luteic acid Uro M5 Uro M6, Uro D Uro C Uro G	Luteic acid Uro M5 Uro M6 Uro C IsoUro A Uro A	[35,36]
*Enterocloster* spp. (*Enterocloster bolteae*, *Enterocloster asparagiformis*, *Enterocloster citroniae*, *Enterocloster pacaense*)	9-dehydroxylase	Uro C Uro M6 Uro D IsoUro A	Uro A Uro M7 Uro G Uro B	[35,36]

**Table 3:** Preclinical and clinical studies evaluating the anti-inflammatory activity of Uro A.

Experimental Model	Doses	Outcomes	Ref.
*Preclinical studies*			
LPS-stimulated murine bone-marrow-derived macrophages (BMDMs)	In vitro: 25 μM or 50 μM for 24, 48 or 72 h.	Downregulation of inflammatory miRNA expression; inhibition of superoxide production; modest attenuation of ROS; reduction in intracellular Ca^2+^ levels and DNA double-stand breaks; downregulation of IL-1β, IL-6, IL-12, TNF-α, NOS2, IFN-γ, TGF-β, IL-10, and IL-2; decrease in TLR4 expression; inhibition of NF-κB; reduction in MAPK activation; suppression of AKT and mTOR stimulation.	Abdelazeem et al. (2021) [40]
Rat articular chondrocytes	In vitro: 1, 7.5, and 15 μM for 2 or 48 h.	Protection against IL-1β-induced cartilage matrix degradation, suppression of the IL-1β-induced-overproduction of MMP9 and ADAMTS4 mRNA and reduction in MMP3 and MMP13 expression in a dose-dependent manner. Dose-dependent prevention of IL-1β-induced degradation of Sox-9, collagen II, and Aggrecan. Decrease in inflammation through the suppression of IL-1β-induced expression of iNOS and COX-2 in chondrocytes at 7.5 and 15 μM. Inhibition of the MAPK and NF-κB pathways thus protecting against IL-β-induced inflammation.	Ding et al. (2020) [41]
Human THP-1-derived macrophages	In vitro: 40 μM for 1 h.	Strong suppression of TNF-α production, induction of the expression of the anti-inflammatory cytokine TGF-β1, inhibition of the NFκB pathway, induction of ERK1/2 phosphorylation.	Bobowska et al. (2021) [42]
RAW 264.7 macrophages		Decrease in NO production and downregulation of iNOS, suppression of pro-inflammatory mediators’ production through the inhibition of the NOX-derived ROS-mediated PI3K/Akt/NF-κB, JNK/AP-1 and p38 signalling pathways.	
Human PBMC-derived macrophages		Stronger inhibition of TNF-α production than in THP-1 macrophages, marked increase in IL-10 receptor surface expression.	
Human primary neutrophils		Suppression of azurophilic granule release.	
4-month-old male C57Bl/6J mice (meniscal destabilization mouse model)	In vivo: 250 mg/kg for 8 weeks.	Reduction in cartilage degeneration and MMP3 levels, alleviation of joint pain and decrease in synovitis and inflammation.	D’Amico et al. (2022) [43]
Human gastric cells (GES-1)	In vitro: 20 μM or 50 μM for 2, 4, 8, 12 or 24 h.	Inhibition of *H. pylori* proliferation and adhesion, attenuation of *H. pylori*-induced inflammation.	Yu et al. (2022) [44]
9–11-week old female Balb/c mice	In vivo: 50 mg/kg every 2 days for 4 weeks.	Alleviation of gastric mucosal inflammation, restoration of IL-10 levels, downregulation of IL-6, IL-8, CXCL1, CCL2 and TNF-α expression.	
14-week-old male C57BL/6 mice (periodontitis mouse model)	In vivo: 20 mg/kg/day via intragastric administration for 2 weeks.	Downregulation of IL-6 and TNF-α expression, alleviation of bone resorption and promotion of collagen fibre reconstruction and repair.	Xia et al. (2025) [45]
8–10-week old C57BL/6 mice, gender was not specified (mouse models of acute lung injury and sepsis-induced inflammation)	In vivo: 50 mg/kg intraperitoneally as a single dose 30 min prior to the induction of injury (LPS administration or CLP surgery)	Downregulation of iNOS, COX-2 and IL-1β and mitigation of sepsis.	Mohsin et al. (2025) [46]
4-week-old female BALB/c mice (OVA-induced acute allergic asthma mouse model)	In vivo: 20 mg/[kg(bw)-d] for 26 days	Alleviation of airway inflammation and inhibition of NLRP3 inflammasome activation via the EGFR/AKT/FOXO3a pathway.	Hou et al. (2026) [47]
9–11-week old female C57BL/6 mice (cyclophosphamide-induced POF mouse model)	In vivo: 41.67 mg/kg of a formulation composed of Uro A, CoQ10, calcium L-5-methyltetrahydrofolate and Vitex agnus-castus extract for 6 weeks	Reverse of cyclophosphamideiec-induced ovarian oxidative–inflammatory shift. Alleviation of inflammation and notable decrease in lipid peroxidation and TNF-α levels. Reduction in IL-1β, IL-6 and other pro-inflammatory cytokine levels.	Dai et al. (2026) [48]
*Clinical studies*			
Healthy middle-aged adults (40–64 years) (NCT03464500)	500 mg or 1 g daily for 4 months.	Reduction in C-reactive protein (CRP), IFN-γ, IL-1β and TNF-α levels.	Singh et al. (2022) [49]
Healthy older adults (65–90 years) (NCT03283462)	1 g daily for 4 months.	Reduction in CRP levels.	Liu et al. (2022) [50]
Resistance-trained male athletes (NCT number was not mentioned)	1 g daily for 8 weeks.	Reduction in CRP levels.	Zhao et al. (2024) [51]
Healthy middle-aged adults (45–70 years) (NCT05735886)	1 g daily for 28 days.	Reduction in IL-2 plasma levels.	Denk et al. (2025) [52]

**Table 4:** Antioxidant activity of Uro A: chemical systems and preclinical studies.

Experimental Test and Model	Doses	Outcomes	Ref.
*Chemical systems*			
ORAC test	In vitro: 4.4 μM–4.4 mM In vitro: not reported	The antioxidant activity measured was 13.1 µmol Trolox equivalents/mg	Cásedas et al. (2020) [54]
DPPH test	In vitro: not reported	IC_50_ = 152.66 μM	
Superoxide scavenging activity	In vitro: 0.5–4 μM	IC_50_ = 5.01 μM	
*Preclinical studies*			
Neuro-2a cells subjected to oxidative stress	In vitro: 0.5–4 μM	Mitigation of oxidative stress, reduction in ROS levels, reduction in lipid peroxidation, enhancement of mitochondrial activity and increase in SOD, CAT, glutathione reductase and glutathione peroxidase activities.	Cásedas et al. (2020) [54]
8-week-old male spontaneously hypertensive rats (SHR)	In vivo: 50 mg/kg intraperitoneally, every 2 days for 4 weeks	Reduction in ROS and serum malondialdehyde and superoxide levels. Increase in mitochondrial SL-SIRT3 levels and SOD2 activity, promoting a reduction in mitochondrial ROS levels in the aorta and mesenteric arteries.	Dai et al. (2026) [55]
Chilled canine spermatozoa	In vitro: 1, 5 μM.	Reduction in malondialdehyde and H_2_O_2_ levels, increase in total antioxidant capacity and enhancement of the activity of GPx, SOD, and CAT.	Mirzaei et al. (2026) [56]

**Table 5:** Enhancement of mitochondrial health by Uro A. Preclinical and clinical studies.

Experimental Model	Dose	Outcomes	Ref.
*Preclinical studies*			
Chilled canine spermatozoa	In vitro: 1, 5 μM.	Reverse in the storage-period-induced loss of MMP and decline in oxygen consumption rate and ATP levels.	Mirzaei et al. (2026) [56]
Porcine parthenotes	In vitro: 0.01, 0.1, and 1 µM.	Enhancement of mitochondrial function and promotion of mitochondrial biogenesis. Upregulation of SIRT1 and PGC-1α levels.	Choi et al. (2026) [59]
*Clinical studies*			
Healthy older humans (NCT03283462)	1 g/day for 4 months.	Reduction in plasma ceramides associated with CVD risk, reduction in acylcarnitines and enhancement of muscle endurance.	Liu et al. (2022) [50]
Highly trained male distance runners (NCT04783207)	1 g/day for 4 weeks.	Reduction in muscle damage and upregulated pathways involving mitochondrial protein expression.	Whitfield et al. (2025) [60]

**Table 6:** Preclinical and clinical studies regarding the cardioprotective effects of Uro A.

Experimental Model	Dose	Outcomes	Ref.
*Preclinical studies*			
24–26-month-old male C57BL/6 mice	In vivo: 25 mg/kg for 2 months	Prevention of age-related decline in diastolic function, and reduction in plasma ceramides.	Liu et al. (2025) [64]
7-week-old Wistar male rats (rat heart failure model)	In vivo: 25 mg/kg for 2 months	Improvement in systolic and diastolic function, enhancement of ejection fraction, decrease in telesystolic volume, improvement in cardiac muscle contractility, and systolic function restoration.	
8-week-old male C57BL mice with induced myocardial ischemic reperfusion (I/R) injury	In vivo: after a standard acclimatization period, mice were administered 1 mg/kg Uro A intraperitoneally, 24 h prior to the surgical operation to induce myocardial I/R injury.	Reduction in serum lactate dehydrogenase and creatine kinase levels and of the infarct size; alleviation of cellular oedema; reduction in myocardial ROS levels.	Su et al. (2024) [65]
11–13-week-old male C57BL/6 mice and H9C2 cardiomyocytes	In vitro: 5 μM. In vivo: 30 mg/kg daily for 4 weeks (intraperitoneal injection).	Attenuation of doxorubicin-induced cardiotoxicity by enhancing PINK1-regulated mitophagy; promotion of Ambra1 expression, which stabilizes PINK1 by inhibiting its degradation via LONP1. Alleviation of doxorubicin-induced cardiac dysfunction both in H9C2 cells and C57BL/6 mice, mitigation of cardiomyocyte contractile dysfunction, myocardial injury and apoptosis. Attenuation of doxorubicin-mediated damages in cardiac function, cardiac atrophy, enhanced fibrosis and reduced cell size.	Wang et al. (2025) [63]
26–28-week-old male C57BL/6 mice fed a High-Fat Diet	In vivo: 50 mg/kg/day by gavage for 20 weeks.	Amelioration of metabolic cardiomyopathy; increase in LC3 and P62 levels; upregulation of the genes Parkin and PINK; increase in autophagic vesicles for the clearance of damaged mitochondria; improvement of cardiac diastolic function; alleviation of cardiac fibrosis and cardiac hypertrophy.	Huang et al. (2023) [66]
*Clinical studies*			
UA non-producers with poor VEF	10 mg or 50 mg daily for 12 weeks.	Improvement in VEF, evidenced by increased Flow-Mediated Dilatation (FMD) scores.	Nishimoto et al. (2023) [67]

**Table 7:** Uro A and kidney disease: preclinical studies.

Experimental Model	Dose	Outcomes	Ref.
High glucose-treated mouse podocytes	In vitro: 10 μM for 12, 24 or 48 h of incubation.	Downregulation of ROS production and anti-apoptotic effects; upregulation of autophagy markers (LC3B, ATG5); increase in nephrin expression.	Kotewicz et al. (2022) [68]
HG-treated HK-2 cells	In vitro: 20 μM	Downregulation of p62 expression; reduction in macrophages infiltration; reduction in pro-inflammatory cytokine levels and decrease in mitophagosomes.	Liu et al. (2024) [69]
6–7-week-old male C57BL/6J mice (DKD mice model)	In vivo: 50 mg/kg/d for 4 weeks by gavage	Attenuation of renal tubular injury and tubulointerstitial inflammation by activation of PINK1/Parkin-mediated mitophagy.	
TCMK-1 and HK-2 cells	In vitro: 50 μM	Downregulation of COM-induced mRNA expression of KIM-1 and NGAL; restoration of PINK1 and Parkin expression, thus inducing mitophagy.	Sun et al. (2026) [70]
9-week-old male C57BL/6J mice (Ca oxalate crystal-induced renal injury mouse model)	In vivo: 10 mg/kg (intraperitoneal) for 7 days	Upregulation of PCK1, enhancement of mitophagy and reduction in calcium oxalate crystal deposition and tubular apoptosis.	
TGF-1-treated HK-2 cells	In vitro: 25, 50, or 100 μM	Alleviation of renal tissue impairment, decrease in macrophage infiltration, downregulation of proinflammatory cytokines (TNF-, IL-6, and MCP-1) expression, attenuation of epithelial–mesenchymal transition by reducing TGF-β1, p-Smad3 and p-38/JNK/ERK, upregulation of the expression of Smad7, and suppression of TGF-β1/Smad and MAPK signalling pathways.	Cheng et al. (2021) [71]
Sprague–Dawley (SD) rats (gender and age were not specified) (UUO rat model)	In vivo: 10, 20, or 40 mg/kg/d for 14 days	Inhibition of TGF-β1/Smad and MAPK signalling pathways.	
6–8-week-old male C57BL/6J mice (UUO-induced fibrosis mouse model)	In vivo: 20, 25 and 30 mg/kg daily by oral gavage for 2 weeks (1 week prior to UUO surgery and 1 week post- surgery)	Improvement of renal damage and fibrosis. Reduction in lipid accumulation and enhancement of fatty acid oxidation. Inhibition of GSK3β/β-Catenin signalling through the activation of GSK3β.	Li et al. (2026) [72]
9-week-old male C57BL/6J mice treated with cisplatin	In vivo: 10 mg/kg of P2Ns-NAR-UA 3 times a week for 4 weeks	Downregulation of IL-6, IL-8, TNF-α. Decrease in neutrophil gelatinase-associated lipocalin, cystatin C, and osteopontin (kidney injury biomarkers). Reduction of tubular damage and renal fibrosis. Upregulation of HO-1.	Wahab et al. (2026) [73]
8-week-old female MRL-lpr mice	In vivo: 10 mg/kg of Uro A and 5 mg/kg of CsA daily for 10 weeks. Urine samples were collected at the 12th and 16th weeks of age.	Improvement of lymphatic and renal function, reduction in urinary protein and serum creatinine levels. Mitigation of inflammation, reduction in lymphocyte infiltration, IgG deposition in kidneys and in IL-6, IL-2, IL-10, TNF-α, IFN-γ, CCL19, CCL7, CCL22, CCL3 and CCL1 serum levels.	Ganugula et al. (2026) [74]

**Table 9:** Uro A’s activity in the gastrointestinal tract.

Experimental Model	Dose	Outcomes	Ref.
6-week-old male C57BL/6J mice with DSS-induced colitis	In vivo: 20 mg/kg/day for 3 weeks	Reverse in weight body loss, alleviation of diarrhoea and haematochezia, increase in colon length, and improvement of colon histological parameters. Alleviation of gut microbiota dysbiosis, decrease in TNF-α, IL-1β and IL-10, and inhibition of the phosphorylation of the MAPK signalling pathway and activity. Restoration of indole-3-aldehyde, indolylpropionic acid, indole-3-lactic acid and L-kynurenine levels, resulting in a reduction in intestinal permeability and an enhancement of intestinal barrier functions.	Ma et al. (2023) [89]
Human neonatal in vitro model of necrotizing enterocolitis	In vitro: 100 μM	Improvement of epithelial barrier function. Decrease in TNF-α and IL-1β transcript levels. Downregulation of NF-κB, NOD-like receptor, TLR signalling and IL-17/Th17. Upregulation of the expression of AhR-dependent detoxifying enzymes.	Sami et al. (2026) [90]
6–8-week-old male and female C57BL/6 mice (DSS-induced colitis mouse model)	In vivo: 20 mg/kg orally every 48 h (on alternate days) starting from day 2 of DSS treatment (or day 0 in non-DSS control groups) until day 12	AhR triggered-NLRP6 activation, and release of IL-18. Promotion of IL-22 production from innate lymphoid type 3 (ILC3) cells. Upregulation of mucin 2 and regenerating family member 3 gamma.	Ghosh et al. (2026) [91]
Intestinal cells isolated from IBD patients.	In vitro: 25 μM	Activation of the intestinal epithelial cells–AhR–NLRP6 axis and increase in IL-18 levels with subsequent induction of IL-22 and of mucin 2 and regenerating family member 3 gamma.	
11-week-old congenic male and female B6N.129S6-Is (19BORCS7-AS3MT) 1Bhk/Mmnc mice (humanized AS3MT mice)	In vivo: 20 mg/kg orally, three times weekly for 10 weeks	Protection of gut barrier integrity, preventing inorganic arsenic-induced intestinal injury and downregulation of tight junction proteins. Decrease in IL-6, TNF-α and IL-1β levels and alleviation of inorganic-arsenic-induced systemic inflammation and gut dysbiosis. Decrease in myeloperoxidase activity.	Ghosh et al. (2026) [92]
7–9-week-old male C57BL/6J mice with D-gal induced liver injury and senescence	In vivo: 5 mg/kg/ day for 8 weeks	Inhibition of phosphorylation of Nur77 and modulation of the hepatic Nur77-MDM2 axis. Downregulation of IL-6 and IL-1β, and restoration of IL-10 levels. Reduction in D-gal-induced hepatocyte inflammation and hepatocyte swelling, structural disorganization, and inflammatory infiltration. Amelioration of D-gal-induced dysfunction and dysregulated lipid metabolism.	Xiao et al. (2026) [93]

**Table 10:** Uro A in metabolic syndrome and diabetes.

Experimental Model	Dose	Outcomes	Ref.
3T3-L1 cells	In vitro: 10, 25, and 50 µM.	Concentration-dependent decrease in lipid accumulation during adipogenesis, enhancement of lipolysis through an increase in free glycerol release, and suppression of lipogenesis by reducing glycerol-3-phosphate dehydrogenase activity. Downregulation of PPARγ, C/EBPα and SREBP-1c. Inhibition of the expression of ACC at 25 and 50 µM, and of adipocyte fatty-acid-binding protein and fatty acid synthase at 50 µM. Downregulation of leptin and adiponectine at 25 and 50 µM.	Berköz et al. (2026) [94]
14-week-old male SPF-grade C57BL/6J mice (HFD-induced obese mice)	In vivo: 20 mg/kg/day for 7 weeks after feeding the mice with HFD	Alleviation of systemic inflammation and amelioration of dyslipidaemia and parameters associated with obesity, such as perinephric and mesenteric fat and triglyceride levels. Increase in HDL-C levels.	Geng et al. (2025) [95]
L6 myotubes under insulin absence	In vitro: 50, 100, 200 µM	Enhancement of glucose uptake through PI3K/Akt and AMPK signalling pathways. Increase in p-Akt and p-AMPK levels and thus induction of GLUT4 translocation from the cytosol to the plasma membrane.	Kondo et al. (2024) [96]
5-week-old male KK-A^Y^/TaJcl mice (T2D model).	In vivo: 100 mg/kg body weight for 3 weeks	Prevention of the hyperglycaemic effects by suppressing the glucose injection-induced increase in blood glucose levels.	
6-week-old male C57BL/6J mice (T2DM mouse model)	In vivo: 25 and 50 mg/kg orally for 9 weeks.	Reduction in blood glucose and serum TNF-α and insulin levels. Inhibition of TNF-α signalling. Improvement of insulin signalling pathway transmission in muscle tissues. Increased expression of genes involved in glycogen synthesis.	Wang et al. (2027) [98]
Primary human myotubes.	In vitro: 50 µM.	Enhancement of basal- and insulin-stimulated glucose uptake. Upregulation of GLUT4.	Wilhelmsen et al. (2025) [97]

**Table 11:** Uro A anticancer properties: preclinical and clinical studies.

Experimental Model	Dose	Outcomes	Ref.
*Preclinical studies*			
SAS and RCB 1015 OSCC cell lines	In vitro: 40 μM	Induction of autophagy and apoptosis, increase in ROS levels, G2/M or S phase cell cycle arrest and antiproliferative effects in a time-dependent manner. Upregulation of p53 and p27, and morphological alterations of cells. Inhibition of the mTOR/AKT/ERK1/2 pathway. Attenuation of invasive potential.	Remadevi et al. (2024) [99]
7-week-old NOD-SCID mice	In vivo: 20 mg/kg daily/vehicle control (10% glucose in water) orally for 12 days	Reduction in tumour volume. Induction of necrosis and apoptosis.	
Tumour-associated macrophages obtained from THP-1 or BMDMs treated with tumour conditioned media. Breast cancer cell lines MDA-MB-231, BT-549, MCF-7 and 4 T1.	In vitro: 5, 10, 25 and 50 μM In vitro: 5, 10, 25 and 50 μM	Downregulation of mRNA expression of the proinflammatory cytokines IL-6 and TNF-α, and reduction in M2 polarization. Promotion of TFEB’s nuclear translocation, induction of mitophagy and prevention of cytoplasmic leakage of nucleic acids, thus reducing macrophage inflammation. Antiproliferative activity and attenuation of migration in breast cancer cells. Inhibition of transcription of genes like c-myc, muc1 and cyclinD1 in MDA-MB-231 and BT-549.	Zheng et al. (2025) [100]
JJN3, U266, RPMI-8226, XG-1 and ARP-1 multiple-myeloma cell lines	In vitro: Concentration range is not specified	Cytotoxicity, inhibition of proliferation and induction of cell cycle arrest. Upregulation of p27, BAX, p53 and p21.	Rodríguez-García et al. (2025) [101]
CD38+ and CD138+ plasma cells from multiple-myeloma patients	Ex vivo: 15 and 30 μM	Reduction in cell viability.	
Bortezomib-resistant (JJN3-R-Btz cells) and bortezomib-sensitive JJN3 cell line	In vitro: Uro A at 15 µM, Btz at 4 nM	Synergistic cytotoxic effect. Upregulation of p53 and downregulation of p-mTOR in JJN3-R-Btz cells. Upregulation of p21 levels in both cell lines.	
Female 6–8-week-old NOD. Cg-Prkdcscid Il2rgtm1Wjl/SzJ (NSG) mice subjected to tail vein injection with JJN3 cells (multiple-myeloma mouse model)	In vivo: 200 mg/kg body weight intraperitoneally 5 times a week.	Inhibition of the proliferation of tumour cells.	
7–9-week-old male C57BL/6 mice (colorectal cancer mouse model)	In vivo: 100/mg/kg daily by oral gavage for approximately 16 days	Enhancement of CD8^+^ T-cell infiltration and cytotoxic activation. Inhibition of tumour growth. Suppression of proliferation, migration and invasion of tumour cells by modulating the AKT/mTOR signalling pathway.	Sun et al. (2026) [102]
*Clinical studies*			
Normal and obese patients (50 to 65 years-old) (NCT04066816)	56 g/day of peeled walnuts for 3 weeks	Increase in serum peptide YY levels; decrease in serum sICAM-1 levels. Downregulation of the expression of vimentin and CD163 in colonic polyp tissue.	Moussa et al. (2025) [103]

**Table 12:** Clinical trials with Uro A supplementation.

Name of the Clinical Trial	Number	Phase	Study Completion
A Randomized, Double-blind, Placebo-controlled Study to Investigate the Efficacy of AMAZ-02 on Muscle Function in Otherwise Healthy Middle-aged, Overweight and Inactive Individuals.	NCT03464500	Not applicable	30 September 2019 (actual)
Open-labelled, Crossover and Randomized Study with Mitopure™ (Proprietary Urolithin A) Dietary Supplementation Compared to Pomegranate Juice Intake: A Bioavailability Study in Healthy Adults (NOURISH).	NCT04160312	Not applicable	31 March 2020 (actual)
A Randomized, Double-blind, Placebo-controlled Trial to Evaluate the Impact of a Daily Dose of AMAZ-02, a Food Derived Ingredient, for 4 Months on Skeletal Muscle Energetics and Function in Healthy Elderly.	NCT03283462	Not applicable	28 October 2020 (actual)
Ellagic Acid, Urolithin and Colonic Microbial Communities Affected by Walnut Consumption.	NCT04066816	Not applicable	8 April 2021 (actual)
A Randomized Double-Blind, Placebo-Controlled Study of the Effects of MPX Capsules on Rising Prostate-Specific Antigen Levels in Alanine/Alanine SOD2 Genotype Men Following Initial Therapy for Prostate Cancer	NCT03535675	Phase 3	3 November 2022 (actual)
A Randomized, Double-Blind, Placebo-Controlled Trial to Evaluate the Effects of Mitopure (Urolithin A) on Skeletal Muscle Function, Iron Metabolism and Endurance Performance in Athletes.	NCT04783207	Not applicable	30 August 2023 (actual)
Impact of Urolithin A Supplementation on Mitochondrial Health of Immune Cells (MitoImmune): a Randomized Trial.	NCT05735886	Not applicable	31 August 2024 (actual)
Evaluation of the Anti-aging Efficacy of One Face Care Cosmetic Product.	NCT06619457	Not applicable	12 December 2024 (actual)
A Comparative Pharmacokinetic Profiles of Five Urolithin A Formulations in Healthy Volunteers: A Randomized, Open-label, Single-dose, Parallel-arm Study.	NCT06362018	Not applicable	14 March 2025 (actual)
Impact of Urolithin A (Mitopure) on Mitochondrial Quality in Muscle of Frail Older Adults.	NCT06556706	Not applicable	25 April 2025 (actual)
Comparison of High-protein Formulation With/Without Urolithin a During a Unilateral Knee Immobilization: A Pilot Proof-of-concept Trial.	NCT05814705	Not applicable	30 April 2025 (estimated)
A Comparative Pharmacokinetic Profiles of Urolithin A Formulations in Healthy Volunteers: A Randomized, Open-label, Single-dose, Parallel-arm Study.	NCT06853197	Not applicable	5 May 2025 (actual)
Urolithin A Supplementation to Improve Endothelial and Cerebrovascular Function in Middle-aged Adults with Obesity.	NCT05921266	Not applicable	14 May 2025 (actual)
Comparative Pharmacokinetics of Urolithin A (Mitopure) Formulations in Healthy Adults: A Randomized, Open-label, Single-dose, Parallel-arm Study.	NCT07231848	Not applicable	28 November 2025 (actual)
Microbiota, Metabolites and Colon Neoplasia.	NCT05195970	Not applicable	30 January 2026 (actual)
PRecision gerOMedicinE: Tailored Healthy agEing With Lifestyle, sUpplements and drugS (PROMETHEUS).	NCT07451496	Not applicable	30 June 2026 (estimated)
Open-labelled Study to Validate Home-based Use of a Health and Wellness Kit (The Mitopure Challenge) to Detect Levels of Urolithin A in Dried Blood Spots After Intake of Mitopure (Proprietary Urolithin A).	NCT04985630	Not applicable	31 August 2026 (estimated)
Radicle Clarity™ 25_RAM: A Randomized, Double-Blind, Placebo-Controlled Direct-to-Consumer Study Assessing the Impact of Health and Wellness Products on Cognitive Function and Related Health Outcomes.	NCT07060898	Not applicable	20 November 2026 (estimated)
Evaluation of Urolithin A and Fisetin on Improving Sleep and Aging Biomarkers in Middle-Aged and Older Adults: A Randomized Controlled Trial.	NCT06990256	Not applicable	30 December 2026 (estimated)
A Randomized Study of Urolithin A vs. Placebo in Patients with Previously Untreated Solid Tumors Receiving Immune Checkpoint Inhibitors.	NCT07161310	Not applicable	March 2027 (estimated)
The Effects of Urolithin A Supplementation on Glucose Metabolism in Healthy Adults ≥ 55 Years Old. A Randomized Triple-Masked Controlled Clinical Trial.	NCT06274749	Not applicable	31 March 2027 (estimated)
A Phase 2 Placebo-Controlled Trial of Urolithin A Supplementation in Men with Prostate Cancer Undergoing Radical Prostatectomy.	NCT06022822	Phase 2	1 May 2027 (estimated)
Polyphenol Metabolism and Personalized Nutrition in Menopause: Metabotypes, Microbiome-virome, and Relationship with Quality of Life and Cardiovascular Risk.	NCT07182370	Not applicable	31 August 2027 (estimated)
Cerebrospinal Fluid Kinetics of Urolithin A in Humans.	NCT07517913	Not applicable	1 October 2027 (estimated)
A Confirmatory, Randomised, Double-Blind, Placebo-Controlled, Parallel Study to Assess the Effects of a Dietary Supplement Containing Urolithin A (Mitopure^®^) on Muscle Strength and Performance in Healthy Middle-Aged Adults.	NCT07231783	Not applicable	31 October 2027 (estimated)

## Data Availability

No new data were created or analysed in this study. Data sharing is not applicable to this article.
